# Ongoing multiparameter unrest at the Montagne Pelée volcano on Martinique from 2019 to 2024

**DOI:** 10.1038/s41598-025-05641-6

**Published:** 2025-07-02

**Authors:** F. R. Fontaine, J.-C. Komorowski, J. Corbeau, A. Burtin, J.-B. de Chabalier, R. Grandin, J. M. Saurel, P. Agrinier, S. Moune, F. Jadelus, D. Melezan, J.-G. Gabriel, C. Vidal, B. Zimmermann, D. Vaton, J. Koziol, J. M. Lavenaire, R. Moretti, A. Lemarchand, T. Labasque, P.-H. Blard, B. Tibari, L. Zimmermann, C. Aubaud, J. Vergne, A. Andrieu, A. Filliaert, E. Chilin-Eusebe, S. Z. Wahlgren, M. Inostroza, J.-P. Métaxian, A. Potier, I. Fernandez, V. Robert, S. Deroussi, G. Carazzo, S. Tait, I. Vlastelic, D. E. Jessop, S. Bonaimé, A. Le Friant, M. Chaussidon, A. Michaud-Dubuy, L. Retailleau, A. Di Muro, P. Allard, C. Satriano

**Affiliations:** 1 Institut de physique du globe de Paris (IPGP), Université Paris Cité, CNRS, F-75005 Paris, France; 2 Observatoire volcanologique et sismologique de Martinique (OVSM), Institut de physique du globe de Paris, 97250 Saint-Pierre, France; 3 Now at Observatoire des Sciences de l’Univers de La Réunion (OSU-Réunion), UAR 3365, Université de La Réunion, CNRS, IRD, Météo-France, Saint-Denis, La Réunion, 97744 France; 4 CNRS, UMR 6524, Laboratoire Magmas et Volcans, OPGC, Clermont-Ferrand, France; 5 Observatoire volcanologique et sismologique de la Guadeloupe, Institut de physique du globe de Paris, 97113 Gourbeyre, France; 6 Dipartimento di Ingegneria, Università degli Studi della Campania “L. Vanviteli”, Via Roma 29, Aversa, I-81031 Italy; 7 Géosciences Rennes, Université de Rennes 1, 35042 Rennes, France; 8 CRPG, CNRS, Université de Lorraine, 54500 Vandoeuvre-lès-Nancy, France; 9 EOST-ITES, Université de Strasbourg, CNRS, UMR 7063, 67084 Strasbourg, France; 10 Millennium Institute on Volcanic Risk Research - Ckelar Volcanoes, Avenida Angamos 0610, Antofagasta, Chile; 11 IRD, UAR IMAGO - LAMA, F98800 Nouméa, France; 12 Observatoire Midi-Pyrénées, 31400 Toulouse, France; 13 Observatoire volcanologique du Piton de la Fournaise, Institut de physique du globe de Paris, La Plaine des Cafres, 97418 La Réunion, France; 14 CNRS, UMR 5276, LGL - TPE, Université Lyon 1, OSUL, Villeurbanne, 69622 France

**Keywords:** Natural hazards, Volcanology

## Abstract

Multiparameter volcanic unrest has been recorded since April 2019 on the Montagne Pelée volcano located on Martinique. There have been only very few periods of seismic unrest since the last magmatic eruption of 1929−1932. This is therefore a rare opportunity to examine its origin. In April 2019 the number of shallow volcano-tectonic (VT) earthquakes increased drastically above the reference monthly rate of 19 VT/month and then exceeded it consistently for several months. Deep (> 10 km) VT events occurred at the onset of the unrest and harmonic tremor was first recorded in November 2020. Continuous Global Navigation Satellite System data reveal that a minor horizontal deformation began around mid-2021. The modeling of these data favors an inflation source located at about 1 km below and slightly SW of the summit, in the area of the hydrothermal system and where most of the shallow VT events are located. Zones of degraded and dead vegetation on the upper flanks of Montagne Pelée were detected with satellite imagery starting in November 2019 and shown to be associated with elevated passive CO_2_ soil degassing. This protracted unrest most likely reflects the ascent of a limited volume of deep magmatic fluids that reinvigorated the shallow hydrothermal circulation.

**Supplementary Information:**

The online version contains supplementary material available at 10.1038/s41598-025-05641-6.

## Introduction

Volcanic unrest is defined^1^ as the “deviation from the background or baseline behaviour of a volcano towards a behaviour which is a cause for concern in the short-term (hours to few months) because it might prelude an eruption”.

The goal of this paper is to document the observations, along with their uncertainty, associated with the ongoing volcanic unrest of Montagne Pelée in the north of Martinique and to provide a conceptual model of the origin of this unrest. This will help to assess whether this unrest phase and the future unrest phases of this volcano could lead to a magmatic or non-magmatic (phreatic) eruption. Depending on its style and dynamics, an eruption of Montagne Pelée, whether it is of non-magmatic or magmatic origin, is likely to have a significant impact on the population^2–4^ environment, and economy, hence requiring preparatory or contingency actions^5^.

After presenting the multiparameter changes recorded by the monitoring network that constitute the most recent phase of unrest at Montagne Pelée, we discuss their origin, a conceptual model to explain the origin of this unrest, and the implications in terms of the likelihood of a new eruption at this hazardous volcano.

## Background

**Fig. 1:**
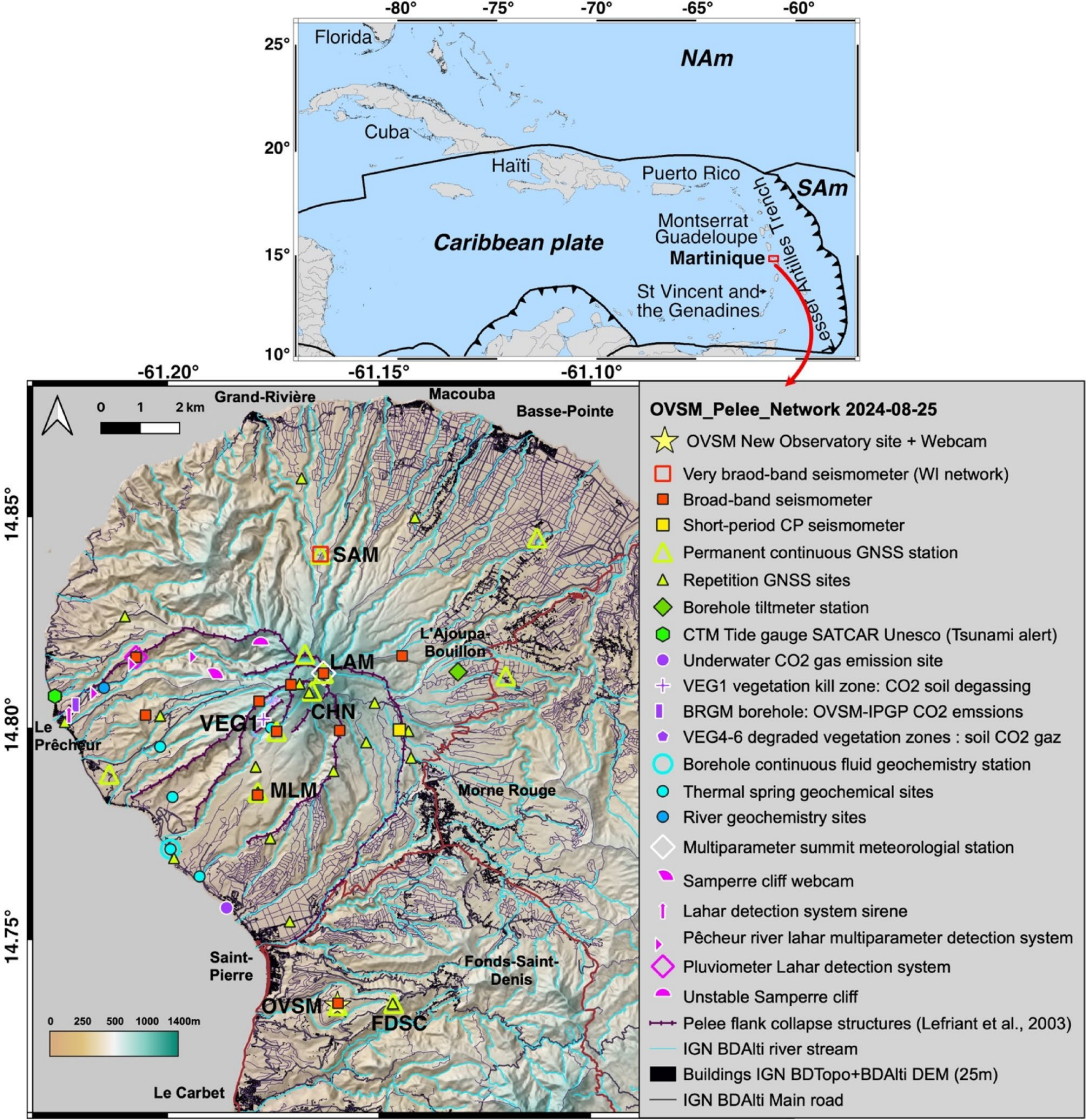
Location of Montagne Pelée volcano on the Caribbean plate and locations of the seismic, ground deformation, and water and gas monitoring networks of the Observatoire volcanologique et sismologique de Martinique (OVSM-IPGP). NAm and SAm are the North American and South American plates. Plate boundaries are from Bird^14^. The smallest picture was generated with GMT software, whereas the main map was produced with QGIS software with a digital elevation model of Montagne Pelée at 25-m resolution taken from IGN.

Montagne Pelée (Fig. 1) is one of the most active stratovolcanoes of the Lesser Antilles subduction zone; it has experienced two to three magmatic eruptions per 1000 years over the last 25,000 years^2,3,6,7^ including three explosive Plinian eruptions in the last 2000 years, in 79 CE^8^ 280 CE^9^ and 1300 CE^10^. All magmatic eruptions are preceded by less intense explosive phreatic eruptions, although phreatic eruptions can also occur without being followed by a magmatic eruption, as in 1792 and in 1851−1852^6,11,12^. There have been four eruptions over the last 250 years^6,13^ including the phreatic eruptions of 1792 and 1851−1852, as well as magmatic dome-building eruptions in 1902−1905 and 1929−1932. With its 29,000 deaths and the devastation of the towns of Saint-Pierre and Morne Rouge, the infamous 1902−1905 eruption was the deadliest eruption of the 20th century^13^.

The reconstruction of the eruptive past^3,6–10^ has shown that eruptions of Montagne Pelée are frequently highly explosive, exhibiting two contrasting eruptive styles. The fragmentation of a gas-rich rapidly ascending magmatic column leads to open-vent Plinian eruption generating abundant tephra fallout of widespread distribution and an associated pyroclastic density current, with a runout of about 10 km^8–10^. Frequent dome-building eruptions can involve the rapid ascent of more viscous non-degassed magma that produces typical dome-collapse pyroclastic density currents but also recurrent phases during which the dome is destroyed by laterally directed explosions that generate devastating high-energy pyroclastic density currents that are highly devastating at a distance of up to 10 km, as best exemplified by the 1902−1905 eruption^2,15^.

The Global Assessment Report on Disaster Risk Reduction 2015 [https://www.preventionweb.net/english/hyogo/gar/2015/en/home/download.html, last accessed August 1, 2024] indicates that Martinique is among the most vulnerable regions in the world to volcanic hazards given its insular context and the proportion of the entire population of the island living 30 km or less from an active volcano. Thus, extensive monitoring of this dangerous volcano is paramount to detect the onset of unrest and the dynamics of its evolution. It is particularly crucial to enhance the ability to identify and interpret monitoring signals that might herald precursory signs of the destabilization of the volcanic system due to magma rise and to evaluate the likelihood of a new phreatic or magmatic eruption.

It is noteworthy to recall the observations that were made during the unrest phase of the devastating explosive magmatic eruption of Montagne Pelée in 1902. As early as 1889, the first signs of renewed activity of Montagne Pelée were noted, with the appearance of a fumarole from a hole in the summit crater of Etang Sec and damaged vegetation in the vicinity^13^. This hole disappeared in 1899, and rich vegetation was observed growing in its place. From at least June 4, 1900, to the beginning of 1902, the number and intensity of fumaroles increased in the Etang Sec summit crater (odors were felt by the population as of February 1902) until April 23, 1902, when the first phreatic explosion with ashfall occurred^11,16^. Summit fumaroles in the area of the 1902−1905 and 1929−1932 lava domes were active until 1970^17^. No fumarolic activity has been observed since 1970. The only surface manifestations of the hydrothermal system of Montagne Pelée are the presence of several thermal springs on the upper and lower flanks of the volcano, with the most important ones being those of the Chaude River, Claire River, Picodo River, and Mitan River, as well as a few hydrothermal submarine springs on the Caribbean Sea coast to the southwest of Montagne Pelée (Fig. 1)^18,19^.

### Actions accomplished in the context of the yellow alert level

Following an analysis of the developing unrest by the Observatoire volcanologique et sismologique de Martinique (OVSM) of the Institut de physique du globe de Paris (IPGP) and its resulting recommendations, authorities set the alert level to yellow (vigilance) on December 4, 2020. Several actions were performed to reinforce and expand the volcano monitoring network (see Supplementary Notes). The OVSM-IPGP initiated in December 2020 the publication of a weekly report that is still ongoing and is complementary to the monthly bulletin. The OVSM-IPGP expanded its nominal communication (see Supplementary Notes) and made significant contributions (hazard evaluation) to the new March 2022 revision of the ORSEC volcano crisis response plan of the Préfecture of Martinique^20^ which was tested in a first volcano crisis exercise on December 7, 2022.

## Results

### Seismicity

**Fig. 2:**
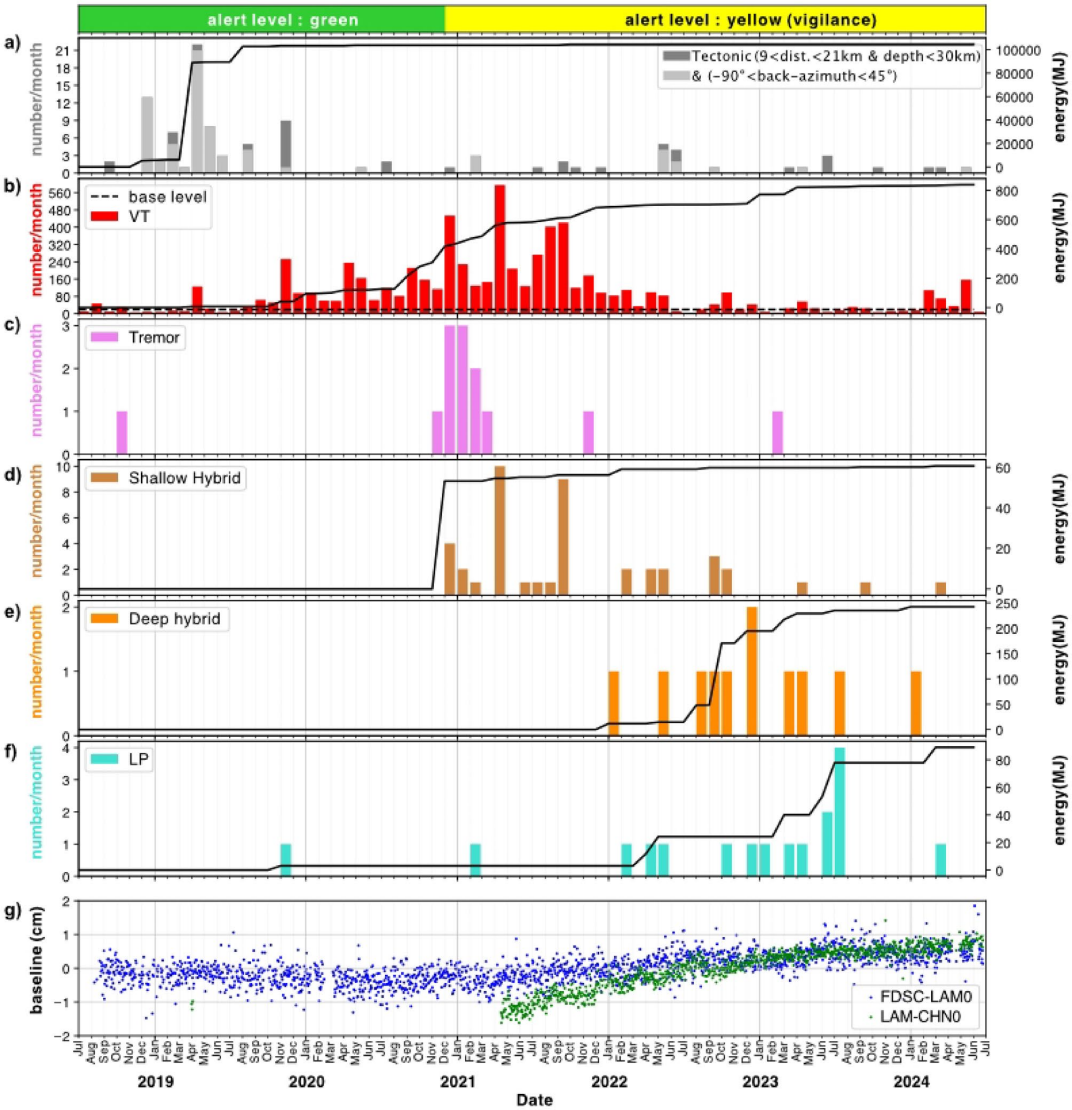
Seismic and geodetic observations from July 1, 2018, to July 1, 2024, at Montagne Pelée. (**a**) Monthly number of detected tectonic earthquakes from a selection of tectonic events occurring in the 9–21 km distance range from the summit of the volcano with depths lower than 30 km and their cumulated energy. The monthly number of tectonic events from this selection characterized by a back-azimuth in the range − 90° to 45° is also indicated in gray for comparison. (**b**) Monthly number of detected VT earthquakes and cumulated energy of VT earthquakes for the same period at Montagne Pelée. (**c**) Monthly number of detected volcanic tremor events. Monthly number and cumulated number of detected shallow hybrid (**d**), deep hybrid (**e**), and LP (**f**) events are also represented. **g**) The evolution of the GNSS horizontal length (i.e., baseline) between the FSDC and LAM and LAM and CHN stations is shown for comparison.

The seismic monitoring network of the OVSM-IPGP consists mainly at the time of writing of 11 permanent three-component broad-band stations and one permanent vertical-component short-period station (Fig. 1). During the month of April 2019, a total of 126 VT earthquakes, occurring in two main day-long swarms and located at relatively shallow depths (up to 4–5 km below the summit), were recorded. This marked a clear increase above the average monthly baseline of VT seismicity (see Methods) (Fig. 2). Starting from November 2019, the seismicity increased systematically every month until it reached a rate of 297 VT/month (using a 28-day moving average) in December 2019, and then a rate of 493 VT/month in December 2020. This constitutes a 25-fold increase in the monthly number of VT events compared to the baseline. The volcanic shallow-depth VT seismicity then reached a monthly maximal rate in April 2021, with 614 VT/month, and remained 5 times above the baseline almost continuously until April 2022. Thereafter, the VT seismicity began to slowly decrease, reaching the baseline threshold in January 2023, but occasional short-lived swarms have repeatedly overpassed the baseline threshold since then (for example, in March, April, and September 2023, as well as from February through May 2024). With 233 VT earthquakes recorded in 2023, the VT seismicity recorded at Montagne Pelée still remains, 5 years after the onset of unrest on April 1, 2019, just barely below the yearly average baseline of 251 VT/year established by the OVSM-IPGP (cf. Supplementary Notes).

The yearly average of the cumulated seismic energy of the located VT earthquakes from January 2015 to January 2019, the reference period for establishing the baseline seismicity rate, is around 26 MJ. Hence, we consider this value as the reference level. The cumulated located seismic energy of VT earthquakes in 2019 (36 MJ) was 38% higher than the reference level. A strong nearly 8-fold increase in the cumulated seismic energy of the located VT earthquakes was then observed in 2020, with 273 MJ. In 2021, this value (252 MJ) was still 10 times higher than the reference level. The cumulated located seismic energy decreased to a value close to the reference level in 2022 (27 MJ) and then increased again in 2023 (75 MJ). Its current value of nearly 8 MJ for the period of January 1, 2024, to June 30, 2024, is below the reference baseline (Fig. 2).

The overall energy of the located VT seismicity between April 1, 2019 and June 30, 2024, is around 670 MJ. Most of the energy was released during the main period of VT activity in 2020 and 2021. However, the largest VT event of magnitude M_d_=2.0 occurred on January 31, 2023, with an energy of 63 MJ, which accounts for 9.4% of the total energy of the located VT events. No VT earthquake was felt or reported, except for an M_d_=0.8 event that occurred during a field expedition of the OVSM-IPGP team at the Chaude River on November 4, 2021.

**Fig. 3:**
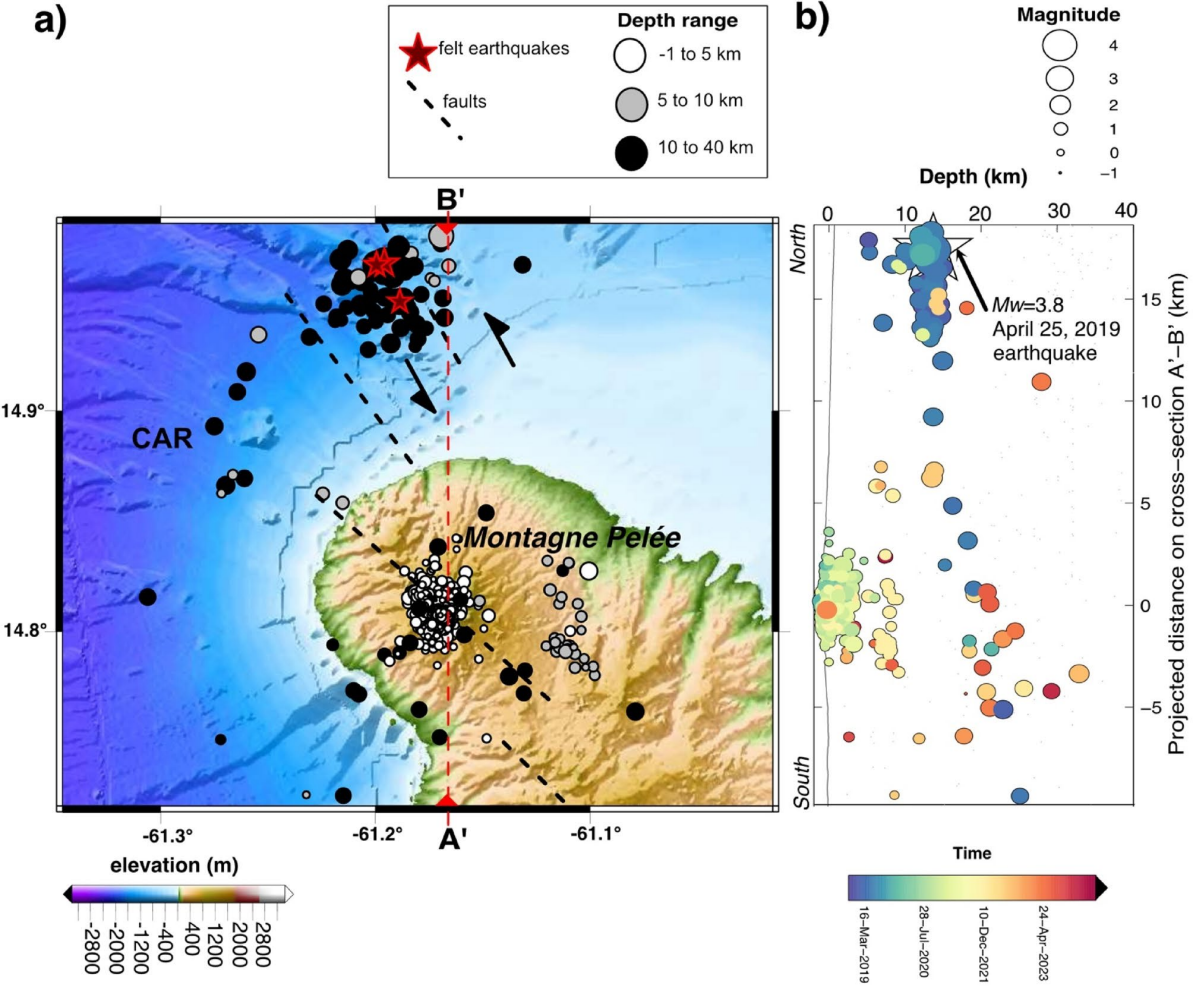
Spatio-temporal migration of seismicity from November 1, 2018, to July 1, 2024. (**a**) Map of the seismicity under Montagne Pelée showing earthquakes localized shallower than 41 km. Tectonic faults are represented^20^. The full focal mechanism of the April 25, 2019, Mw = 3.8 earthquake (Fig. S5) is composed of a 41.1% double-couple component, a 31.3% compensated linear vector dipole (CLVD) component, and a 27.5% volumetric component. It is compatible with a rupture on a quasi N-S striking strike-slip fault plane in transtension north of Montagne Pelée with a large non-double-couple component. The map was developed with GMT software 4.5.7 and a digital elevation model of Montagne Pelée from the shuttle radar topography mission (30-m resolution) and bathymetry from Ryan et al.^22^. CAR: Caribbean plate. (**b**) N-S profile of the seismicity during this period. Note that the scale of the circles, which is relative to the event magnitude, is not the same as in (**a**). The color scale provides an indication of the event occurrence time.

Most of the VT seismicity corresponds to a repeater-type earthquake called a VT-1 earthquake, which is located in the area of the hydrothermal system of the volcano at a maximum depth of about 1.0 ± 0.7 km below the surface (Figs. 3 & S2). This VT-1 family has been observed since at least the 1970s^21^ and is still presently observed (July 2024). However, nine new families of VT repeater earthquakes have been detected based on a peak correlation with a master event since the 2019 unrest, with each having specific spatio-temporal characteristics, including two families located within the 6–10 km depth range that began to be recorded in November/December 2021 (Table**S2**).

Since 2019, the OVSM-IPGP has also recorded a few long-period (hereafter called LP, see Methods) earthquakes and hybrid earthquakes (see Methods) that were not present in the OVSM-IPGP catalog before 2019 (Fig. 2d and f). Among the 54 hybrid events recorded, 33 have been located, and their depths show a bimodal distribution, with 25 shallow events (named hybrid in Fig. 2) at less than 5 km below the surface and eight deep events (named deep hybrid in Fig. 2) at depths between 20 and 35 km. The epicenters of the shallow hybrid events are less than 2 km from the summit of the volcano (20 out of 25). They appeared in December 2020 and mostly occurred during the most intense period of VT activity. The deep hybrid events are more widely distributed (epicentral distance from 1.5 to 15 km) and occurred later in 2022 and 2023. The overall energy released by all located hybrid events (shallow hybrid plus deep hybrid events) was around 174 MJ, with 66% corresponding to the deep hybrids (Fig. 2). The total energy released by hybrid seismicity corresponds to 40% of the total VT seismicity energy.

Among the 17 LP events detected since 2019, nine can be reliably located. They are all deep events located at around 20 km depth, except for one located at 37 km depth. Their epicentral location varies from 1 to 16 km from the volcano summit. Except for two events in November 2019 and February 2021 classified with uncertainty, the LP events all occurred in 2022 and 2023, with a peak of activity in July 2023, which also occurred for most of the deep hybrid events (Table**S1** & Fig. 2). The overall energy released by the located LP events between April 1, 2019 and June 30, 2024, was around 89 MJ, which represents 12% of the total VT seismic energy.

In addition, hours-long tremor-type signals were recorded on November 8 and 9, 2020 (Figs. 2c & S4). These signals are characterized by a continuous harmonic signal with a fundamental frequency around 1.8 Hz (Fig. S4) and the corresponding first overtones (harmonics) and bursts of minutes-long periods of higher energy. A few other episodes of tremors occurred up to March 2021. The last two tremor episodes were observed in November 2021 and February 2023. We note that the tremor events mostly occurred during or following periods of high energetic VT activity.

Excluding tremor events, it is noteworthy that a non-negligible proportion of the total seismic energy for the located events (933 MJ) over the period from April 1, 2019, through June 30, 2024, was released by low-frequency seismicity (shallow hybrid, deep hybrid, and LP located events), which accounted for 28% of the total located seismicity, whereas the remaining 72% was released by VT located seismicity.

###  Volcanic edifice deformation

A network of ten permanent Global Navigation Satellite System (GNSS) stations is operating on the volcano. We detected a slight anomaly in the deformation of the volcanic edifice that started in mid-2021 (Fig. 2g) by analyzing the GNSS measurements of the Montagne Pelée network covering the period from November 1, 2018, to July 1, 2024 (Figs S7 and S8). According to the various physical models carried out by the IPGP team, this slight deformation of the upper part of the volcanic edifice (a few millimeters in one year) could correspond to a source of inflation located about 1.0 ± 0.3 km deep below and slightly SW of the summit, above the hydrothermal system. We note the similarity of the source depth with the source of the recurrent VT-1 earthquakes. The volume of the source was estimated to be from 49,000 ± 8,000 m^3^ based on a Mogi model (see Methods), to 89,000 ± 40,000 m^3^ based on a point compound dislocation model (pCDM) modeling (see Supplementary Notes, Methods).

The source of ground deformation can be interpreted as an inflation process caused by the weak pressurization of the volcanic system caused by the rise of small volumes of magmatic fluids (gas and/or liquid) in relation to the volcanic unrest of Montagne Pelée since April 2019. The simplicity of the mechanical models used (homogeneous and elastic medium in particular) and the fact that the measured displacements are very small, with a signal-to-noise ratio that is also quite low (especially vertically), give this observation an intrinsic uncertainty in terms of its interpretation. However, this first observation of the occurrence of a near-field shallow-depth deformation signal at Montagne Pelée is significant and important in the context of the current unrest.

### Phenomenology (field observations): fractures, gas emissions (ground and sea)

**Fig. 4:**
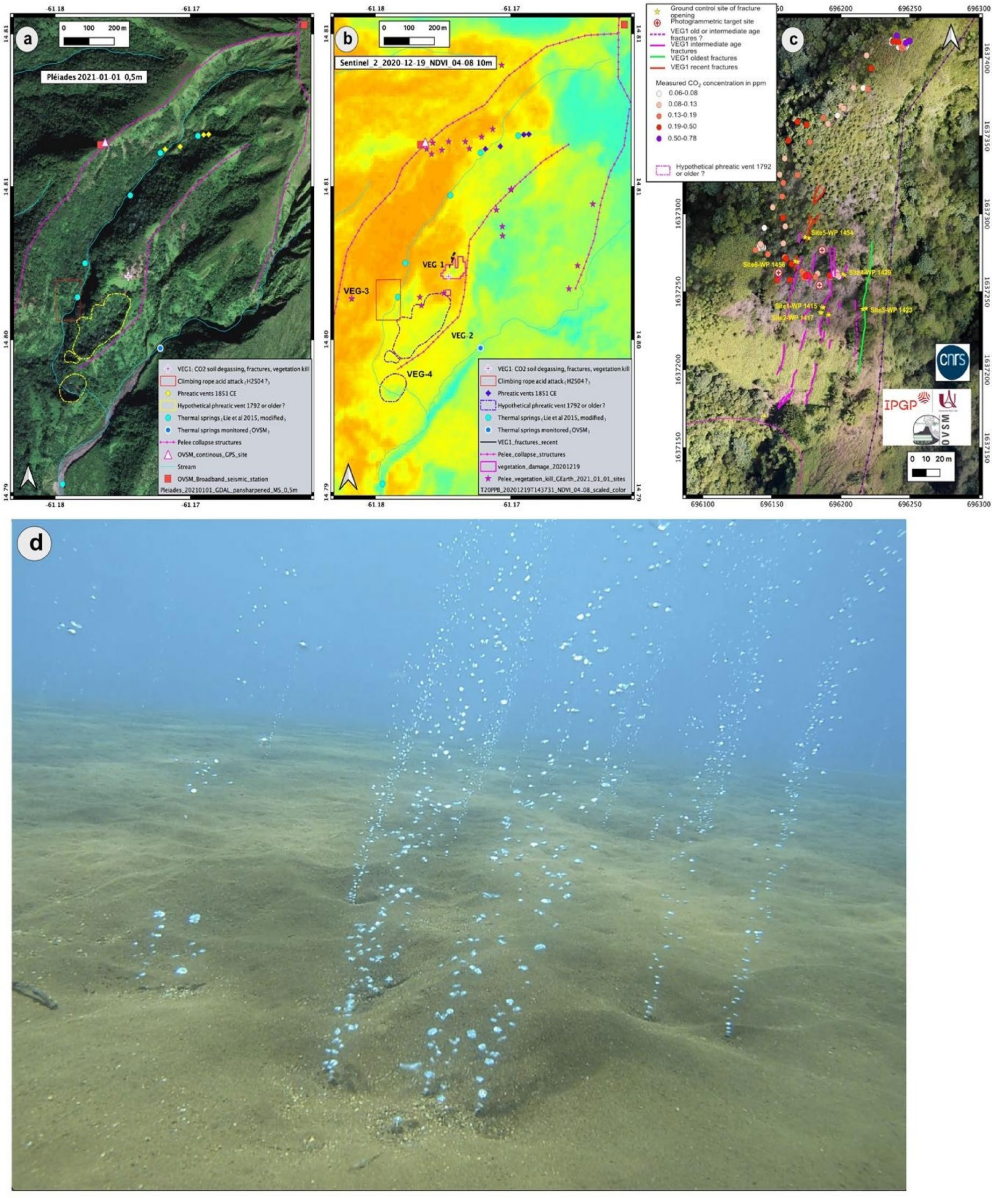
Observations of passive gas emissions. (**a**) Picture from Pleiades (acquired on January 1, 2021) of degraded, browned, and dead vegetation zones and location of the CO_2_ anomalies. The map was produced by the authors with QGIS software version 3.12.0 (https://download.qgis.org/downloads/). (**b**) NDVI map showing the extension of the VEG-1 to VEG-4 zones. The NDVI index provides an estimation of vegetation vigor and therefore its level of degradation, from orange (not degraded) to yellow (slightly degraded) to green (highly degraded), determined by the analysis of Sentinel 2 satellite images of the VEG-1 zone from December 19, 2020. Contains modified Copernicus Sentinel data 2020. This map was produced using the QGIS software version 3.12.0. (**c**) Measured CO_2_ concentration in ppm obtained with a DrägerX-Am-5600 sensor on February 8, 2021, at the VEG-1 site. Photograph (orthoimage) was obtained on March 2, 2021, from a field survey with DOMDRONE. This photograph was taken by the OVSM-IPGP with technical aid of DOMDRONE and it was modified with QGIS software version 3.12.0. Map of fractures was determined from observations on March 5, 2021. (**d**) Submarine gas emission. Picture was taken by the OVSM-IPGP on August 12, 2021. It illustrates the ascent of bubble trains at sea from the seabed at a site located between Saint-Pierre and Le Prêcheur at a depth of less than 11 m.

A zone of strongly degraded, leafless, and dead vegetation called VEG-1 was initially detected visually on December 26, 2020 (Fig.4a, b and c) on the southwest flank of Montagne Pelée, above the confluence of the upper Claire River and the Chaude River. Observatory staff confirmed the distal visual observations during an overflight with the civil protection helicopter on December 29, 2020. Thereafter, an immediate retrospective analysis of satellite imagery readily available on Google Earth dating back to 2004, showed that this zone of degraded and dead vegetation started to appear on satellite imagery between November and December 2019 and that it was clearly visible as of January 2020 (Fig. S9). A field expedition on the ground on February 8, 2021, by the OVSM-IPGP, with the support of the fire and rescue department (STIS) and the civil protection helicopter, confirmed that this total degradation of the vegetation (Fig. S10**)** is associated with the presence of diffuse and passive soil degassing of carbon dioxide (CO_2_, an odorless and colorless gas) from the ground, with concentrations (up to 7800 ppm) largely above baseline values (ca. 400 ppm) (Figs. 4c & S13). On March 21, 2024, a field expedition by OVSM-IPGP staff, with support from the civil protection helicopter, confirmed an elevated CO_2_ flux from passive soil degassing in other areas called VEG-4 and VEG-6, with values between 45 and 84 g/m^2^/day (see Fig. S13) measured with a West Systems accumulation chamber. The last measurements performed on July 30, 2024, in the VEG-4 area reached a maximum value of 179.9 g/m^2^/day.

In the center of the most deteriorated vegetation zone, fractures 0.2 to 0.6 m wide and 2 to 3 m deep are visible on the ground and can be followed for several tens of meters (Figs. 4c & S11). These fractures were formed during different periods in the past. However, the sharpest clear fractures, linked to the degraded vegetation that is bent over them, may have formed very recently, although the observatory staff currently have no precise temporal information. There are no fumaroles or water vapor or gas visibly escaping from any orifice or crack, and there is no thermal anomaly associated with these fractures.

An elevated CO_2_ flux from passive soil degassing has been commonly documented in other volcanic zones^23–25^. This area on Montagne Pelée is located (Fig. 4b and c) at a distance less than 200 m from the upper Claire River where the presence of hydrogen sulfide (H_2_S, smells like rotten eggs) has been reported in the scientific literature for several years^16^. It is also in the vicinity of where canyoners reported in 2019 and 2020 that their fixed descending rope had been partly dissolved by acids, such that it broke into shreds (Ankanionla-Madinina, personal communication); in the vicinity of thermal springs that originate from the hydrothermal system of the Montagne Pelée; and in the vicinity of the sites of the 1792 and 1851−1852 phreatic eruptions^26^ (Fig. 4b and c).

### Monitoring vegetation degradation from passive CO_2_ soil degassing emissions from remote sensing

The monitoring of the Normalized Difference Vegetation Index (*NDVI*) (see Methods and Supplementary Notes) made it possible to identify the first degraded vegetation zone, VEG-1 (Fig. 1), in November 2020 (Fig. S9) and other degraded areas (VEG-2, VEG-3, VEG-5, VEG-4, VEG-6) in the vicinity of the VEG-1 zone (Fig. S12). The initial VEG-1 zone has now been revegetated. We have not been able to regain access to this remote area. Thus, we do not know whether this is the result of opportunistic vegetation that is more resilient to passive soil CO_2_ degassing below a critical threshold or whether, in this area, the CO_2_ flux has effectively diminished to levels acceptable for the vegetation. The VEG-2 zone has continued to progress towards the south on the eastern rim of a probable old phreatic explosive crater, and the VEG-3 zone has progressed slightly to the east. The VEG-4 zone **(**Figs. 4b & S13**)** is well marked and was visited on the ground on March 21, 2024 (see above); we measured elevated CO_2_ passive ground emission levels. A new zone called VEG-6 started to develop in early 2024 and is now clearly visible on satellite imagery (Fig. S13).

### Geochemistry of water and gas from thermal springs and nearshore gas emission

A zone of emission of gas bubbles from the seabed (Fig. 4d) was observed at the mouth of the Pères River, less than 11 m deep, following the testimony of a fisherman that was reported to the observatory staff at the end of June 2021. The quantitative monitoring of this zone of submarine degassing by the OVSM-IPGP began in August 2021. The date for the onset of these underwater fumarole emissions is unknown. However, according to several testimonies, this diffuse degassing could have existed for several decades in this area. An area of 1570 m^2^ was observed in February 2022 that was characterized by the presence on the sandy seabed of many points from which gas bubble trains are emitted, rising continuously to the surface, with a variable flux rate. The sampling of this gas with “Giggenbach”-type bottles^27^ revealed that these gas bubbles consist essentially of CO_2_. This nearshore, underwater CO_2_ degassing is associated with a weak thermal anomaly (maximum value of 2°) and a slightly acidic *pH* (6.25 instead of 8.12 on September 15, 2021). The isotopic analyses of stable carbon isotopes expressed as δ^13^*C* values (defined by ((^*13*^*C/*^*12*^*C*_*sample*_)/(^13^*C/*^12^*C*_*standard*_)−1)×1000) determined from samples taken in July 2022 from these nearshore gas emissions (*δ* ^13^*C = −5.31‰ PDB*) and from samples collected in 2021 and 2022 from the Chaude River (*δ* ^13^*C = −1.40‰* on September 8, 2021, and *δ* ^13^*C = −2.30‰* on May 5, 2022, for thermal spring number 4) confirm that they are both dominated by CO_2_ of magmatic origin. However, the starting date of this magmatic input is unknown. Helium isotopic ratios expressed as *R/Ra* values (where *R* is the ^*3*^*He/*^*4*^*He* measured in the sample and *Ra* is the atmospheric ratio) of thermal springs ranged from 7.08 ± 0.22 to 7.59 ± 0.23 (1σ uncertainty) in 2021−2023. These results suggest a dominant magmatic input. New analyses from March 2024 show broader values, with *R/Ra* ranging from 5.76 ± 0.14 to 7.74 ± 0.19 (1σ uncertainty), confirming a dominant magmatic contribution. At a greater distance from the volcano (6 km), helium in underwater nearshore gas emissions at Saint-Pierre showed in March 2023 an isotopic signature with a greater contribution in helium ^4^He of crustal origin, with *R/Ra* ranging from 5.75 ± 0.18 to 5.97 ± 0.18 (1σ uncertainty) (see the monthly report of June 2023 from the OVSM-IPGP and supplementary information in the file 41598_2025_5641_MOESM1_ESM.xlsx for further details). New analyses of seawater gas from Saint Pierre from March 2024 show lower values, with*R/Ra*between 3.85 ± 0.09 and 5.07 ± 0.12 (1σ uncertainty). Dissolved gases from a water sample of the hottest thermal spring (SC4) of the Chaude River show temporal variation, with enrichment in both ^4^He (from 302% in September 2021 to 1080% of saturation in August 2022) and CO_2_.

The measured δ^13^*C* values are in the field of volcanic gas emissions from volcanoes in the Lesser Antilles arc, marked by inputs of magmatic volatiles^28^. The first isotopic analyses of helium from samples taken in August and September 2022 from gas emissions of the magmatic system of Montagne Pelée indicate that above the magmatic conduit, there is helium degassing of an essentially magmatic mantle origin (Fig. S14).

The enrichment in both ^4^He and CO_2_ is likely mainly due to the fact that a magmatic source as a crustal source is unlikely in the case of a stratovolcano, and indeed the predicted values of *R/Ra* from mixing models between three end-members, the atmosphere, the Mid-Oceanic Ridge Basalt (MORB), and the crust, show a dominant source of helium coming from the upper mantle (see Methods & Fig. S14). We observed for the first time at Montagne Pelée an increase in the concentrations of He and CO_2_ from 2021 to mid-2022 and then a decrease in these gases in 2023 and 2024. This trend could be interpreted as a degassing pulse followed by near-state conditions.

An increase in the temperature of thermal springs from the Chaude River was reported between 1960 (53 °C) and 1967 (83 °C)^17^. This was then followed by a decrease until 2009. The temperature readings of thermal springs from the Chaude River do not exceed the maximum value (36.8 °C) measured by the observatory in March 2009. This maximum temperature has not been exceeded since 2009.

## Discussion

###  Hypotheses to explain the observed changes in volcanic seismicity since 2019

Different sources and processes seem to be at the origin of the recorded volcanic seismicity:

1. There is no clear evidence that the increased VT seismicity in April 2019 could be linked to the gradual increase of tectonic seismicity on a regional scale^29^ which could perturb the stress field of the volcano. A total of 28 events were identified and localized at a depths from 9 to 18 km between December 2018 and August 2019 (see Supplementary Notes). Such distal seismicity has been considered a precursor of several eruptions at long-dormant (> 20 years) stratovolcanoes^23,24^ such as the 1995 Soufriere Hills eruption on Montserrat^25^ and the 1991 eruption of Mount Pinatubo^26^. Deep magmatic fluid injection and/or circulation, as suggested by the much deeper VT seismicity, may exert pressure transients on the nearby environment surrounding the transcrustal magmatic system, which could induce seismic energy release on tectonic features such as a pre-existing left-lateral fault. The distal seismicity was associated with earthquakes with double-couple mechanisms occurring on pre-existing faults. The motion on a roughly N-S striking strike-slip fault plane in transtension, such as in the Dominica Channel, may favor the upward motion of magma beneath the Dominica Channel^20^.
2. Volcanic earthquakes observed between 15 and 30 km below sea level below or in the vicinity of Montagne Pelée could result from the migration of supercritical crustal fluids at overpressure and magma fluids at depth below the deep magma storage zone postulated approximately between 12 and 16 km deep^3^.
3. On the seismicity map (Fig. 2), few VT earthquakes are located between depths of 5 and 10 km (VT-8 earthquakes near Ajoupa-Bouillon). These earthquakes are localized about 10 km off-center from the main magmatic conduit below the summit, in the depth range of the shallowest magmatic storage system, which could be from 5 to 8 km beneath the volcano summit according to petrologic data^3,28^ or from 7 to 12 km (2 ± 0.5 kbar, assuming a density of volcanic rock of 2.2 g/cm^3^) based on phase equilibrium studies^30^; it may also be associated with magmatic fluid migration. The volume located beneath the summit at depths between 6 and 9 km below sea level is characterized by rare earthquakes and may correspond to a magmatic storage zone.
4. Hypocenters located between 0.2 and 5 km below sea level could be bound to fluids of the supercritical deep hydrothermal system (> 374 °C and > 221 bar) in overpressure and/or magma above the shallowest magma storage area. The maximum depth of the shallow seismicity may be related to the brittle-ductile boundary, which could be located about 5 km below sea level.
5. From 0 to 0.5 km above sea level, where we observe the most frequent category of VT earthquakes and several hybrid events, the overpressured fluids (< 374 °C and < 221 bar) of the superficial hydrothermal system could also induce shallow seismicity in the volcanic edifice, sometimes inducing the oscillation of fluids in nearby fluid-filled cavities or faults.

The deep hybrid and LP events, with a depth range varying between 18 and 37 km, are located just above or below the Moho, estimated at about 26 km depth^31,32^. These deep earthquakes, with a low frequency content, could be generated by the movement of pressurized fluids (magma, gas) feeding the deepest magmatic storage reservoir, which can induce seismic events on fractures in some brittle parts of the lower crust or upper mantle.

**Fig. 5:**
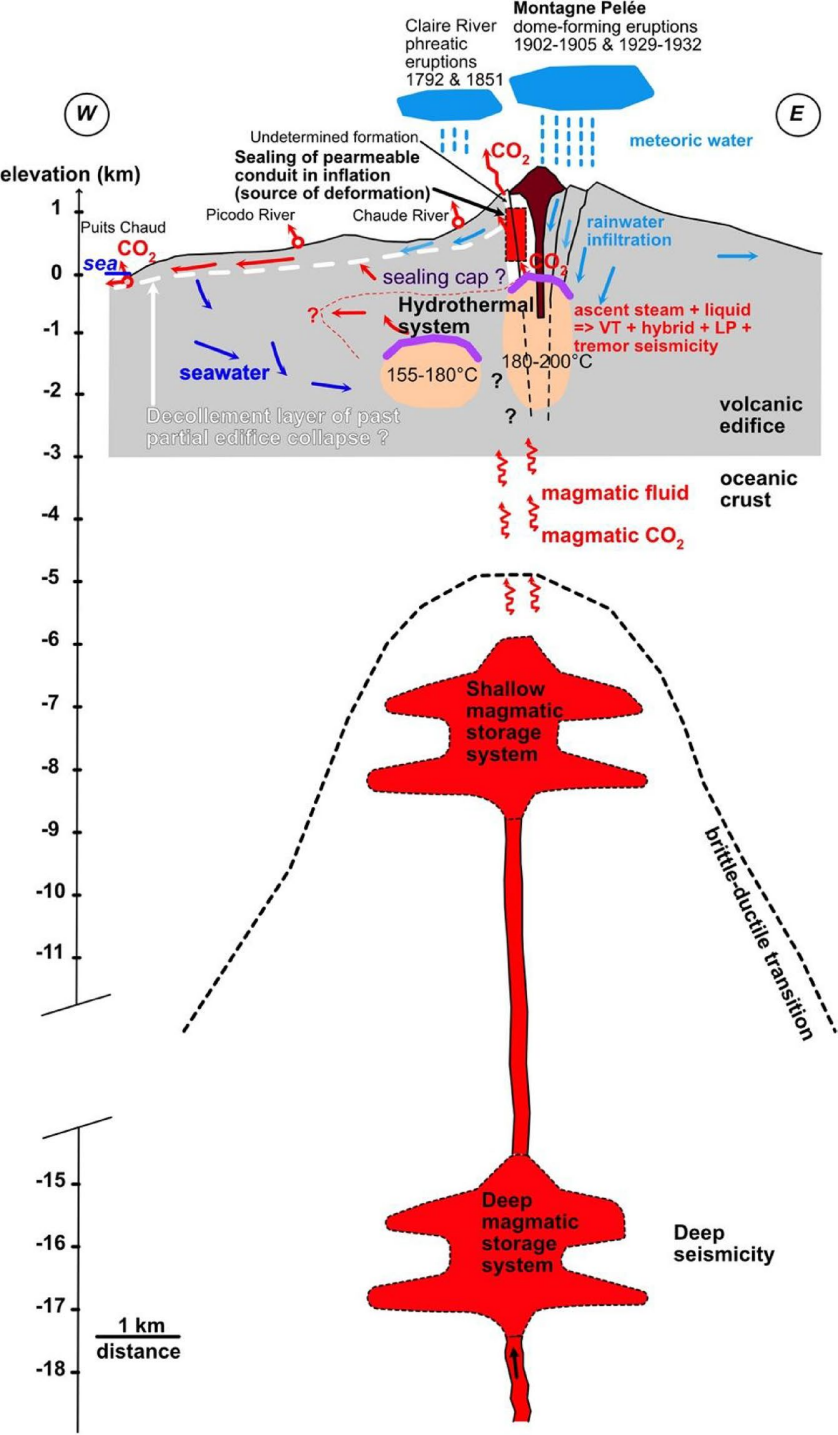
Conceptual model of Montagne Pelée magmatic and hydrothermal system. Modified from Gadalia et al.^18^ with data taken from Le Prieur et al.^33^, Lalubie^34^, Le Friant et al.^35^, Boudon et al.^2^, Zlotnicki et al.^36^, Martel et al.^30^, and Boudon and Balcone^3^.

From the observations presented above, our preferred model to explain the origin of the volcanic unrest of Montagne Pelée is illustrated in Fig. 5. Deep hybrid and LP events (18 to 37 km depth) suggests a deep magma input in a deep magmatic storage zone. This input may be followed by magmatic fluid and CO_2_ migrations towards the surface. Magma migration from a deep magmatic storage zone towards a shallow magmatic storage zone can be hypothesized based on the described seismicity, but not with certainty. The described seismicity alone might indicate a lack of clear evidence for magma migration. However, this uncertainty is reduced by the use of other observations, such as the isotopic analyses of helium that indicate the presence of the degassing of helium, of upper mantle origin, above the magmatic conduit. Therefore, joint observations of the deep seismicity (i.e., deep hybrid and LP events) and isotopic analyses of helium at the Montagne Pelée volcano support the hypothesis of magmatic migration towards a shallow magmatic storage zone. Magmatic fluid and CO_2_ migration from this shallow reservoir to the surface is supported by the increase in the shallow VT seismicity; the volcanic edifice deformation, interpreted as an inflation process caused by the weak pressurization of the volcanic system caused by the rise of small volumes of magmatic fluids; and measurements of magmatic CO_2_ at the surface in underwater fumaroles and as dissolved gas in thermal springs on the flanks of Montagne Pelée. The increase in superficial volcanic seismicity may be related to a perturbation of the hydrothermal system. The tenuous volcanic tremor signals may be associated with the migration of hydrothermal fluids in the volcanic edifice. The top of the hydrothermal system may be variably sealed and was submitted to overpressure, as suggested by ground deformation observations. The degraded vegetation and measurements of magmatic CO_2_ at the surface show the upward motion of gas to the surface.

**Fig. 6:**
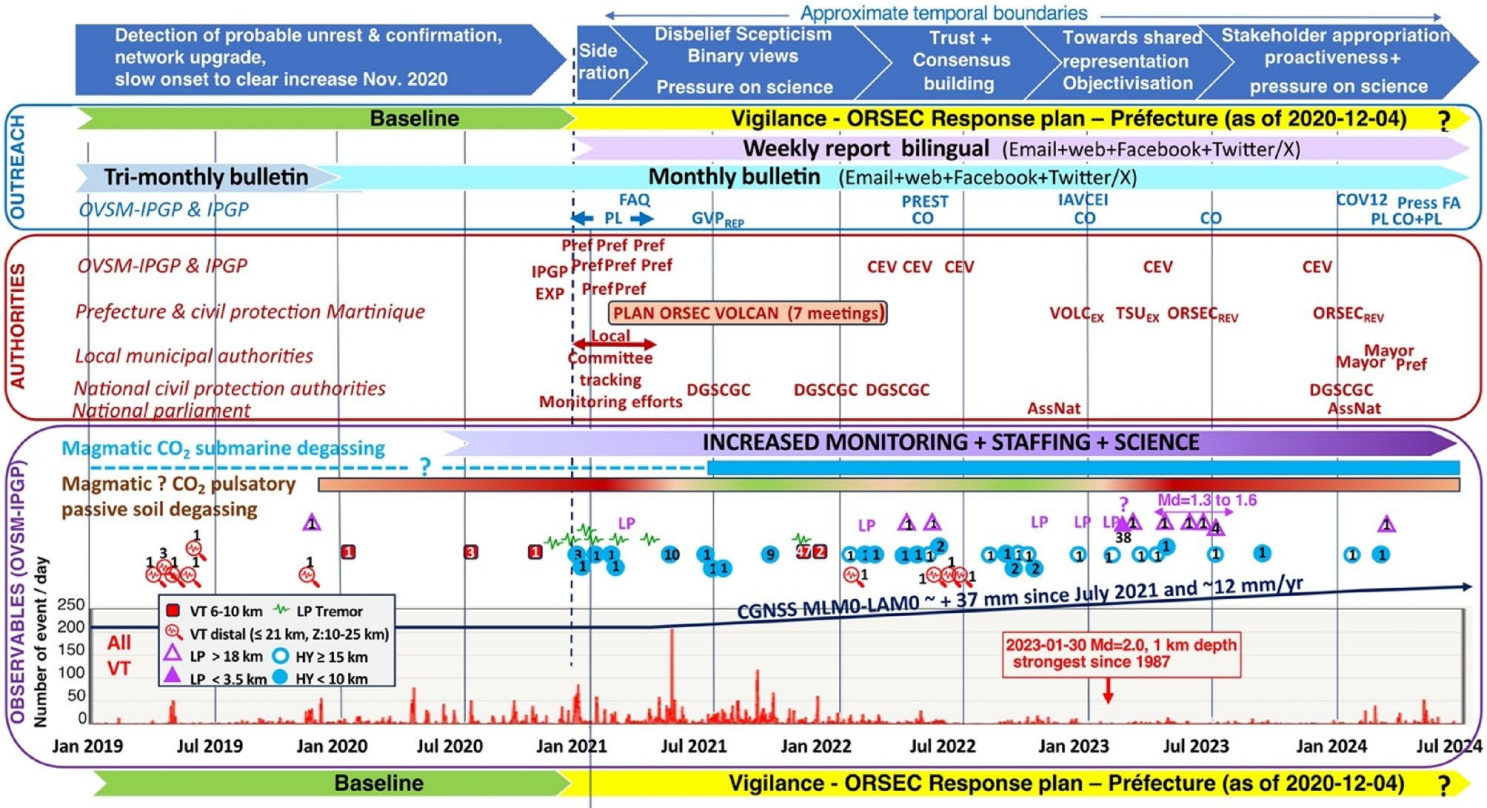
Chronology of the volcanic unrest of Montagne Pelée with observations and actions performed by the OVSM-IPGP between December 1, 2018 and July 1, 2024.

Several communication and outreach actions were undertaken to provide these observations and interpretations to both the authorities and the public (Fig. 6).

## Conclusion

After a period of 87 years since the last magmatic dome-building eruption of 1929−1932, the most significant multiparameter unrest phase of Montagne Pelée was detected as of April 1, 2019. It is non-eruptive, at the time of writing (July 2024), and although it is still ongoing, it is now proceeding at a much-reduced level since 2022. The identification of the nature of the onset of the unrest phase is important to better address possible future eruptive scenarios. A significant increase in the shallow VT seismicity was recorded starting in April 2019. This was followed in 2020 by the occurrence of tremors, then by tremor and hybrid events in 2021, and finally by continuing hybrids associated with deep hybrid and deep LP events in 2022 that have continued to occur occasionally in 2024. Thus, this reflects a pattern of seismicity that deepened from top to bottom through time and increasingly involved deep seismicity with a low frequency content below magma storage zones (15 km), in addition to shallow high-frequency VT seismicity. Such top-bottom seismicity has been previously described at several volcanoes^23^. Two other important observations are the following: (1) low-frequency shallow-source tremor events and shallow-depth (< 5 km) hybrid events occurred primarily during the period of the most intense high-frequency VT shallow-depth seismicity between 2020 and 2021; and (2) the period of low-frequency deep seismicity (> 15 km) corresponds to a period of much reduced shallow high-frequency VT seismicity.

These seismic observations can be reconciled by a model in which aseismic deep magma input of limited volume into the crustal magmatic storage zones of Montagne Pelée, located between 6 and 16 km depth, triggered an increase in the flux of heat and magmatic mantle-sourced CO_2_ into a sealed hydrothermal system. This process favored the pressurization of the shallow hydrothermal system, which triggered shallow VT seismicity associated with a shallow source of inflation. Prolonged shallow VT seismicity is related to a network of connected paths and stressed rocks in the upper crust that depressurized the system and triggered the downward propagation of decompression that enhanced the slow upward movement of deep pressurized magmatic fluid of limited volume from the deep upper mantle region into the crustal reservoirs (6–16 km) and towards the surface, a process that became visible as a burst of located deep low-frequency seismicity (> 15 km) and magmatic CO_2_ mantle-sourced degassing. Over the period of unrest, top-bottom mechanism has enhanced the connectivity of the surface to the areas of crustal magma storage^23^ a process that needs to be carefully monitored given that final connectivity through this depth zone is required for and often detected prior to the onset of eruptive activity^23^. Both the source of edifice inflation and the main locus of the VT hypocenters are associated with fluid migrating from the deep hydrothermal system to more shallow zones. Several zones of strongly degraded and dead vegetation have been tracked with satellite imagery and visually since November 2019 on Montagne Pelée’s SW flank. These zones fluctuate, but new zones appear. At least two of these zones were shown to be associated with passive soil CO_2_ degassing without fumaroles. They are located near active thermal springs and the site of the 1851 phreatic eruption vents.

The ongoing multiparameter unrest of Montagne Pelée underscores that significant changes in the behavior of the hydrothermal-magmatic system of Montagne Pelée have occurred but that they, so far, have not led to an escalation in the nature and amplitude of the unrest. It is impossible to determine whether the current decrease in the intensity of the unrest will eventually lead to a return to baseline activity for an extended period of time or whether the unrest could resume and eventually evolve into more critical conditions at the volcano. Roman and Cashman^23^ underscore that shallow VT seismicity may not reflect initial conduit formation nor the onset of magma storage zone chemical disequilibrium and that these processes can occur initially aseismically at depth or long before the escalation of seismicity just prior to eruption. Hence, the extensive monitoring of this dangerous active volcano is paramount to detect the onset of multiparameter unrest and the dynamics of its evolution. It is particularly crucial to enhance the ability to identify and interpret monitoring signals that might herald precursory signs of (1) a chemical destabilization of the magma storage zones due to magma ascent from depth; and (2) enhanced connectivity between the surface and the magma storage zones. Given that the timescales from magma reservoir destabilization to eruption can be typically short, on the order of a few weeks to 1−2 years^3,15^ this is paramount to improve the assessment of the likelihood of new magmatic unrest.

However, preparing for a hypothetical uncertain yet possible near-future eruption of Montagne Pelée, whether it is phreatic or involves magma ascent, requires the widespread synergetic, consensual, timely, and long-term mobilization of all actors who are part of the societal response to the risks associated with potential eruptive activity in a populated small-island environment.

## Methods

### Monitoring networks of Montagne Pelée

The monitoring networks of Montagne Pelée (Fig. 1), operated by the OVSM of the IPGP, are composed of a seismic network, a ground deformation network, and a thermal spring and gas monitoring network. Presently, the seismic network is composed of 11 permanent three-component broad-band stations, one permanent vertical-component short-period station and two accelerometers. The permanent ground deformation network consists of 10 permanent Global Navigation Satellite System (GNSS) stations. A temporary deployment of 22 GNSS stations was performed in March and April 2021 for more than 10 days. In 2022, 14 temporary GNSS stations were deployed for more than 6 days from April 24 to May 20, and 22 temporary GNSS stations were installed with more than 15 days of acquisition from September 10 to December 19. In 2023, a temporary deployment of GNSS stations was also accomplished in September and October.

Six permanent tiltmeters were also installed at three different depths in a borehole at one site. The tilt determined by tiltmeters is affected by strong-magnitude earthquakes. Temperature changes can also induce variations in inclination, as it is sensitive to changes in atmospheric pressure and temperature^37^. At 30 m, the sudden variations in inclination that can be observed are linked to strong earthquakes. There is no clear visible trend in the long-term variation of the inclination from March 2017 to October 2022^38^. At 60 m, the slope observed since June 2021 could be explained by a change in the instrumental drift of one of the two components of the tiltmeter. These data do not show in 2022 any clear deformation of the Montagne Pelée edifice associated with intrusion or the significant movement of magma at depth. No data were available in 2023 due to a problem with the acquisition system.

The gas monitoring network consists of four sampling sites used for the monitoring of the physico-chemical properties (temperature, conductivity, water level and chemical composition) of thermal springs and a submarine nearshore fumarole and one permanent station for the real-time borehole measurement of the fluid geochemistry in a distal thermal spring. The OVSM is also in charge of the maintenance of a lahar monitoring system installed on the Prêcheur River^39^. As for many volcanological observatories, the different networks operated by the OVSM-IPGP have evolved over time in terms of the number of sites, type of instrumentation, and technologies for data transmission and management, including following the present unrest. This has led to variations in signal detection thresholds. However, to the first order, the detection thresholds have remained relatively stable since 2022.

### Earthquake detection

The earthquake detection was manually processed and validated at the OVSM, a volcanological and seismological observatory of the IPGP. This long-running procedure has been assisted since 2020 by a template-matching detection for volcanic seismic events. To define a given volcano-tectonic earthquake template, the first stage of processing consists of waveform association to highlight seismic repeaters. We use for this purpose the REDPy software^40^ where all volcanic earthquakes are cross-correlated to each other and family association is conducted with a correlation level of 0.7.

This preliminary repeater catalog is then manually checked to remove families with limited occurrences (< 10). The second stage is a test of the preliminary templates, where for each class, all associated waveforms are normalized and stacked to increase the signal-to-noise ratio. These latter templates are then used to scan the previous seismic dataset following a template-matching approach (back to 2013). A new catalog of repeater detections is ultimately used to build the final template waveforms by again stacking all the occurrences. We have then defined a MASTER waveform for a given VT family of earthquakes.

A template-matching scheme was applied to seismic records from five seismic broad-band stations located near the volcano summit (LAM, PBO, MPLM, GBM, and SCH2) from 2013 to 2024. Seismic signals are filtered in a 1–20 Hz frequency band to enhance most seismo-volcanic signals. Finally, a 3.5 s sliding window, starting before the first *P*-wave arrival, is used to continuously scan the seismic signal^41^. Template matching is achieved when the signal correlation between a MASTER waveform and the continuous signal is greater than or equal to 0.6, a value determined by trial and error. This was initially done in post-processing to complete the database, and it is now carried out in quasi-real time.

### Earthquake location and magnitude

For earthquake location, we use the NonLinLoc software package^42^ and a modified velocity model^43^. The magnitude was computed using the duration magnitude^44^ excepting for the three felt tectonic earthquakes from the Dominica Channel for which the local magnitude^45^ is estimated from the vertical component. Full moment tensor inversion is also used to compute the moment magnitude *M*_*W*_ of the largest tectonic earthquakes from the Dominica Channel.

### Baseline of VT seismicity under Montagne Pelée

Since 1980 and the existence of modern monitoring networks (Fig. 1), the volcanic seismicity under Montagne Pelée, detected within a crustal extension cylinder with a radius of 9 km centered on the volcano’s summit and encompassing its morphological inland expression, has been typically very low, with a few tens of earthquakes recorded per year on average^21^ (Fig. S1). Between January 1, 1995, and January 1, 2013, 584 volcano-tectonic earthquakes (hereafter called VT earthquakes) were identified in the manual catalog, with a median value of 10 VT earthquakes per year, a mean of 32 VT earthquakes per year and two particular high values: 106 VT earthquakes in 2006 and 208 VT earthquakes (i.e. the maximum value) in 2012.

An increase in VT seismicity was recorded from 2013−2014, with a total of 3095 events located at depths between 1 and 1.4 km below the summit of the volcano, with a maximum magnitude M_d_ of 2.2. This shallow VT seismicity may be related to a period of unrest of the volcanic system associated with perturbations of the shallow hydrothermal system that may have been enhanced by stress and dynamic permeability perturbations^46^ caused by a M_W_ 6.5 regional earthquake that occurred during this VT sequence.

Between the January 1, 2015, and April 1, 2019, a total of 1069 VT earthquakes were recorded, resulting in a mean annual rate of 251 VT/year, which corresponds to a mean monthly rate of 19 VT/month. The rate of 19 VT/month, or 251 VT/year, is presently considered by the OVSM-IPGP as the baseline activity of Montagne Pelée^47^ between the two most recent periods of unrest, namely 2012−2014 and 2019−2024, and it is the baseline rate retained hereafter for comparison.

### Classification of LP, hybrid earthquakes and volcanic tremors under Montagne Pelée

This classification is mostly based on the frequency content of seismic records at a group of stations on the volcano. VT earthquakes show high-frequency signals (between 5 and 30 Hz) and *P* and *S* phases in the raw (i.e., without filtering) seismograms. LP events do not show *S* phases, and they are characterized by lower frequencies (0.5–5 Hz) than VT earthquakes. Hybrid events have mixed characteristics, between those of VT and LP events, with high-frequency signals present above 5 Hz^48^. Volcanic tremors are continuous seismic signals showing low frequencies, mainly in the range 0.5−5 Hz^49^. Harmonic tremor has a similar appearance, with a fundamental frequency around 1.8 Hz (Fig. S4), and it corresponds to first overtones (harmonics). Non-harmonic tremors show more irregular signals. The frequency content was determined using the following process: the spectra of the vertical component was determined with an Fast Fourier Transform (FFT), and we created a spectrogram for each seismic event using the SWARM (Seismic Wave Analysis/Real-time Monitoring) software (https://volcanoes.usgs.gov/software/swarm/ Version 3.2) and ObsPy, which is a Python toolbox for seismology^50^. We used an overlap of 75% and a typical window length of 60 s for the spectrograms. The sensor instrumental response was not removed, as it is supposed to be flat between 0.5 and 50 Hz. The classification of deep hybrid and LP events is challenging, as it mostly depends on the high-frequency content of the signal, which varies with the event-station distance and with the variable scattering and absorption attenuations at depth. This means that some events classified as LP events could be deep hybrid events, and vice versa. However, the similar period of occurrence and similar depth range of the deep hybrid and LP events may indicate that they have a common origin.

### Full moment tensor inversion

For the single tectonic earthquake from the Dominica Channel with a magnitude *M*_*Lv*_ higher than 4 on April 25, 2019, we conducted a full moment tensor inversion from the seismograms recorded at 11 three-component broad-band stations (Fig. S5) in order to determine the focal mechanism for this earthquake. This event was the single event providing a stable solution with the moment tensor inversion. We used the ISOLA (ISOLated Asperities) software package^51^. The signal-to-noise ratio was computed and used to choose a suitable frequency. The seismic waveforms were bandpass filtered between 0.10 and 0.15 Hz. The time window length was restricted to 245.76 s, covering the full waveform for all seismic stations. We used a source rate time function with a Dirac delta function, the 1D velocity model^43^ and the *v*_*P*_*/v*_*s*_ ratio, quality factors, and density, as detailed in a previous study^29^. Green’s functions were computed using the discrete wavenumber method^52,53^. The optimum solution of the moment tensor was obtained using a least-squares inversion and a space-time grid search for the centroid position and time. The best solution was calculated from the correlation value between the synthetics and the observed seismograms. The correlation value is equal to the squared value of the variance reduction. We fixed the centroid latitude and longitude from the location provided by the OVSM-IPGP public notice for the felt earthquake associated with this event and only searched for its depth and time. The best depth was searched for in a depth range from 3 km below the epicenter to 25 km, with steps of 1 km. The optimum time was determined by shifting the seismograms around the hypocenter time with a step of 0.06 s. The jackknife method was applied to assess the uncertainties of the inversion results (Fig. S6) by repeatedly removing one distinct seismic station from among the group of stations at each inversion in order to test the inversion stability^51^.

We determined the *ε* and *k* values^54^ to describe the source type. *ε *is obtained from the eigenvalues of the deviatoric moment tensor, with *ε*= -*λ*_*2*_/max(|*λ*_*1*_|,|*λ*_*3*_|), where *λ*_*1*_,*λ*_*2*_, and *λ*_*3 *_are the eigenvalues of the deviatoric moment tensor and *λ*_*1*_≥*λ*_*2*_≥*λ*_*3*_. A pure double-couple mechanism is characterized by *ε* = 0 whereas a pure CLVD corresponds to *ε* = −0.5 or *ε* = 0.5. The second parameter *k* is defined as the relative contributions of the isotropic and deviatoric components. *k* is calculated from *k*=*M*_*ISO*_/(|*M*_*ISO*_|+*M*_*DEV*_), where *M*_*ISO *_and *M*_*DEV *_are the isotropic and deviatoric moments, respectively. The earthquake that occurred on April 25, 2019, is characterized by *ε*~−0.22 and *k*~−0.30. This mechanism therefore has a large non-double-couple component, which is well constrained in the inversion (Fig. S6). This magnitude *M*_*W*_ = 3.8 earthquake in the Dominica Channel does not seem to be associated with a change in stresses significant enough to induce volcanic seismicity 15–20 km away in Montagne Pelée. No evidence of deformation associated with a deep magmatic intrusion was detected with the GNSS network on Montagne Pelée following this event.

### Numerical modeling of deformation

We conducted two types of numerical modeling of ground motion displacement measured at GNSS stations: Mogi modeling and point compound dislocation model (pCDM) modeling^55–57^. For Mogi modeling^58^ the displacement is assumed to be caused by a pressurized spherical cavity at depth in an elastic half-space. Both methods are operational in the WebObs system^56^ used at the OVSM-IPGP. The deformation codes used for modeling are available at https://github.com/IPGP/deformation-lib.

### Search for thermal anomaly

A search for a thermal anomaly on the flanks of the volcano was conducted using a thermal camera during helicopter overflights of the deteriorated vegetation zone. Additionally, we reviewed the archive of ASTER thermal image granules (https://ava.jpl.nasa.gov/) but found no signs of a thermal anomaly, possibly due to the relatively small dimensions of the affected zone with respect to the pixel size.

Regular measurements of temperature are conducted by the OVSM/IPGP team at the submarine gas emission site, at thermal springs, and the Prêcheur River^47^. Temperature measurements are performed for submarine gas emissions with a Tinytag AQUATIC 2 TG-4100 (https://www.geminidataloggers.com/data-loggers/tinytag-aquatic2/tg-4100) and Tinytag Plus 2 TGP-4020 submersible data loggers, with 1 measurement taken every 10 s. A portable WTW Multi3620 IDS with a WTW SenTix 940 pH/temperature electrode and a WTW TetraCon 925 conductivity/temperature probe is used for the thermal springs, and the temperature is determined continuously at the Puits Chaud borehole with a PT1000 probe.

### Monitoring vegetation damage caused by volcanic gas with *NDVI*

The *NDVI* is a dimensionless index that is determined by using the contrast between the near-infrared (NIR) reflectance *ρ*_*NIR*_ and the red (Red) reflectance *ρ*_*Red*_. It is calculated from georeferenced multi-spectral images (airborne or satellite images):

1 $$\:{\text{NDVI}}=({\uprho}\text{}_{NIR}-{\uprho}\text{}_{Red})/({\uprho}\text{}_{NIR}+{\uprho}\text{}_{Red})$$

This method has been used on volcanoes to detect and track the impact of volcanic degassing on the vigor of the vegetation with satellite imagery^59–61^. The monitoring of the *NDVI* was carried out based on the analysis of Sentinel 2 satellite images (ESA, CNES, CNRS, 10 m resolution on the first identified vegetation zone, VEG1) and Planet images^62^ (3 m resolution, Imagery © 2021−2025 Planet Labs Inc.). We have tracked, since November 2019 on Sentinel-2 imagery and also since September 2021 with Planet and Pleiades (Pléiades © CNES (2021), Distribution AIRBUS DS) imagery, the evolution of these degraded and vegetation kill zones (Figs S12 & S13). The data were analyzed graphically and visualized with QGIS 3.12.0 software^63^.

### Geochemistry of water and gas emissions

We measured, for the VEG-1 degraded vegetation zone, the CO_2_ concentration in ppm with a calibrated DrägerX-Am-5600 sensor. For VEG-4, the CO_2_ flux was measured using a WEST Systems accumulation chamber following standard methods^64^. The isotopic analysis of carbon isotopes (δ^13^*C* values) was performed using samples taken in July 2022 from nearshore underwater gas emissions and from samples of the Chaude River collected in 2021 and 2022. Analyses of dissolved gases (^4^He and CO_2_) from water samples of the hottest thermal spring of the Chaude River were also conducted. To estimate the δ^13^*C* values of the dissolved inorganic carbon (δ^13^*C*_DIC_) from water samples, we used the analytical protocol detailed in Assayag et al.^65^.

Analyses of the helium isotopic ratios were also conducted for samples of the Chaude River SC4 thermal spring, as well as for samples of gas from the submarine fumarole. ^4^He/^20^Ne ratios were estimated with a quadrupole mass spectrometer, and ^3^He/^4^He ratios were analyzed with an SFT Thermo mass spectrometer. Helium isotopic ratios were corrected for atmospheric contamination assuming that all neon is atmospheric, following Sano et al.^66^.

The results of dissolved gas analysis (Fig. S15) show the presence of two types of groundwater on Montagne Pelée. First, we have recent surface waters in chemical equilibrium with the atmosphere. These are dominated by N_2_, O_2_, and Ar (with some Ne), while CO_2_ and He are very poorly concentrated in the atmosphere. Second, we have older waters, which have percolated into a deeper medium; they are isolated from the atmosphere and have therefore lost a significant part of their dissolved atmospheric gases, and they are also marked by an input of magmatic gases. The latter are mainly CO_2_ and He for Montagne Pelée. The contents of magmatic Ar, Ne, and N_2_ are very low. In the binary diagrams, the Prêcheur River and Puits Chaud are marked by seawater, Forage 2 is dominantly marked by atmospheric gases (N_2_, Ar, Ne, O_2_), and SC4 is the most enriched in magmatic gases (CO_2_, He).

Figure S16 shows the concentrations of He and CO_2_ at the hottest SC4 Chaude River thermal spring and the CO_2_/He ratio as a function of time. Given that the signals are not too disturbed by problems of analysis (long storage time between sampling and analysis) and emergence (degassing), we observe, to the first order, an increase in He and CO_2_ from 2021 to mid-2022. Since mid-2022, we have observed an overall decreasing trend in the concentrations of these gases. This bell-shape pattern starting in 2021 could be interpreted as a degassing pulse. Indeed, from 2021 to mid-2022, the data show a significant general trend (with a factor of 3 increase in the concentration of He and 30% variation in the concentration of CO_2_, which is the most concentrated gas and therefore the most easily measurable).

## Electronic supplementary material

Below is the link to the electronic supplementary material.

Supplementary Material 1

Supplementary Material 2

## Data Availability

All seismic events located by OVSM/IPGP are available on-line through webservices at the IPGP Data Center: http://ws.ipgp.fr. The raw seismic data are available from the EPOS-FRANCE infrastructure, https://seismology.resif.fr and the raw GNSS data are available at the EPOS-GNSS infrastructure, http://volobsis.ipgp.fr/. Pleiades images are available upon request to AIRBUS DS, https://www.intelligence-airbusds.com/geostore/.
