# Supplementary material for: Ongoing multiparameter unrest at the Montagne Pelée volcano on Martinique from 2019 to 2024
Source: Sci Rep. 2025 Jul 2;15:23189. doi: 10.1038/s41598-025-05641-6 (PMC12223141; doi:10.1038/s41598-025-05641-6)
Supplement: Supplementary file 2 — Supplementary Material 2 [file 41598_2025_5641_MOESM2_ESM.docx]

# Supplementary information to the paper:

# **Ongoing multiparameter unrest at the Montagne Pelée volcano on Martinique from 2019 to 2024**

F.R. Fontaine^1,2,3^*, J.-C. Komorowski^1^, J. Corbeau^1,2^, A. Burtin^1^, J.-B. de Chabalier^1^, R. Grandin^1^, J.M. Saurel^1^, P. Agrinier^1^, S. Moune^1,4,5^, F. Jadelus^1,2^, D. Melezan^1,2^, J.-G. Gabriel^1,2^, C. Vidal^1,2^, B. Zimmermann^1,2^, D. Vaton^1,2^, J. Koziol^1,2^, J.M. Lavenaire^1,2^, R. Moretti^1,5,6^, A. Lemarchand^1^, T. Labasque^7^, P.-H. Blard^8^, B. Tibari^8^, L. Zimmermann^8^, C. Aubaud^1^, J. Vergne^9,1,2^, A. Andrieu^1,2^, A. Filliaert^1,2^, E. Chilin-Eusebe^1,5^, S. Z. Wahlgren^1,2^, M. Inostroza^1,5,10^, J.-P. Métaxian^1^, A. Potier^1,2^, Fernandez, I. ^1,2^, V. Robert^1,11^, S. Deroussi^1,4^, G. Carazzo^1^, S. Tait^12^, I. Vlastelic^1,4,5^, D.E. Jessop^1,4,5^, S. Bonaimé^1^, A. Le Friant^1^, M. Chaussidon^1^, A. Michaud-Dubuy^1,4^, L. Retailleau^1,13^, A. Di Muro^14^, P. Allard^1^, C. Satriano^1^

(1) Institut de physique du globe de Paris (IPGP), Université Paris Cité, CNRS, F-75005 Paris, France, frfont@ipgp.fr

(2) Observatoire volcanologique et sismologique de Martinique (OVSM), Institut de physique du globe de Paris, 97250, Saint-Pierre, France.

(3) Now at Observatoire des Sciences de l’Univers de La Réunion (OSU-Réunion), UAR 3365, Université de la Réunion, CNRS, IRD, Météo-France, F-97744 Saint-Denis, France.

(4) CNRS, UMR 6524, Laboratoire Magmas et Volcans, OPGC, Clermont-Ferrand, France.

(5) Observatoire volcanologique et sismologique de la Guadeloupe, Institut de physique du globe de Paris, 97113, Gourbeyre, France.

(6) Dipartimento di Ingegneria, Università degli Studi della Campania "L. Vanviteli", Via Roma 29, Aversa I-81031.

(7) Géosciences Rennes, Université de Rennes 1, 35042 Rennes, France.

(8) CRPG, CNRS, Université de Lorraine, 54500 Vandoeuvre-lès-Nancy, France.

(9) EOST-ITES, Université de Strasbourg, CNRS, UMR 7063, 67084 Strasbourg, France.

(10) Millennium Institute on Volcanic Risk Research - Ckelar Volcanoes, Avenida Angamos 0610, Antofagasta, Chile.

(11) IRD, UAR IMAGO - LAMA, F98800 Nouméa, France.

(12) Observatoire Midi-Pyrénées, 31400 Toulouse, France.

(13) Observatoire volcanologique du Piton de la Fournaise, Institut de physique du globe de Paris, La Plaine des Cafres, La Réunion, France.

(14) CNRS, UMR 5276, LGL – TPE, Université Lyon 1, OSUL, Villeurbanne, France.

**Supplementary Figures**

*
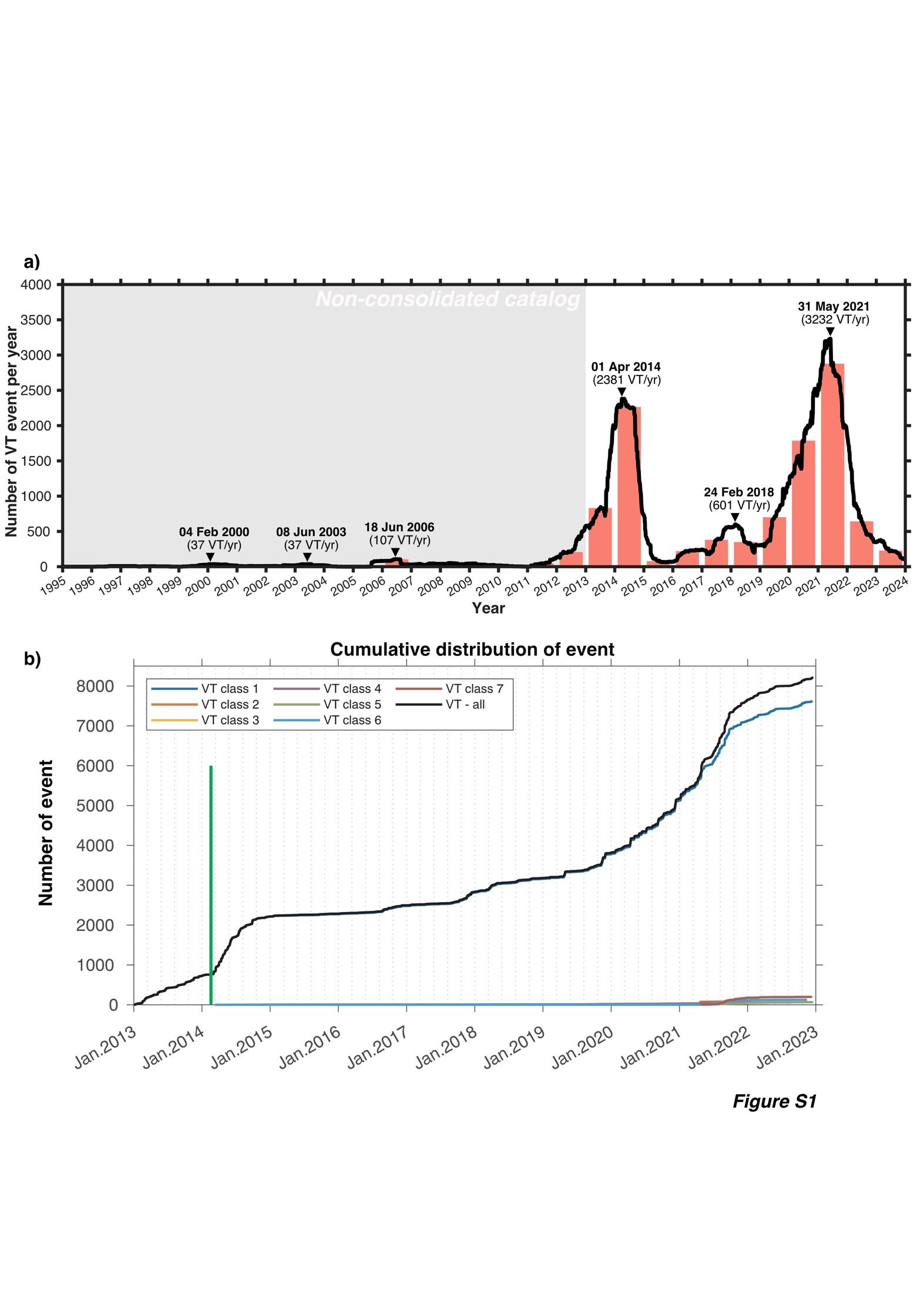
*

***Figure S1.*** *Temporal variation of VT earthquakes under Montagne Pelée. a) Evolution of the yearly number of VT earthquakes recorded at the OVSM IPGP from January 1, 1995, to December 31, 2023 (data from WebObs/OVSM). In red, the number of earthquakes recorded in each calendar year (from January 1 to December 31). In black, the average number determined over 365 days, with the maximum values reached (date and rate). The shaded area indicates data that is not yet consolidated because it does not take into account automatic detections. Figure modified from the OVSM 2023 annual report*^1^*. b) Increase in the cumulative daily number of VT type 1 earthquakes, observed through an automatic search for VT earthquakes detected at the LAM seismic station since January 2013. A template matching approach was used. The contribution of the VT*-*2-7 families in the cumulative number of VT earthquakes is also represented. The analysis focuses on a specific category of VT earthquakes repeating over time since at least 1976*^2^*, which reflects the trend in the overall activity at Montagne Pelée. The green vertical line shows the time of the magnitude (M_W_) 6.5 tectonic earthquake felt on February 18, 2014, at around 200 km east of Martinique, at a depth of 11 km.*

*
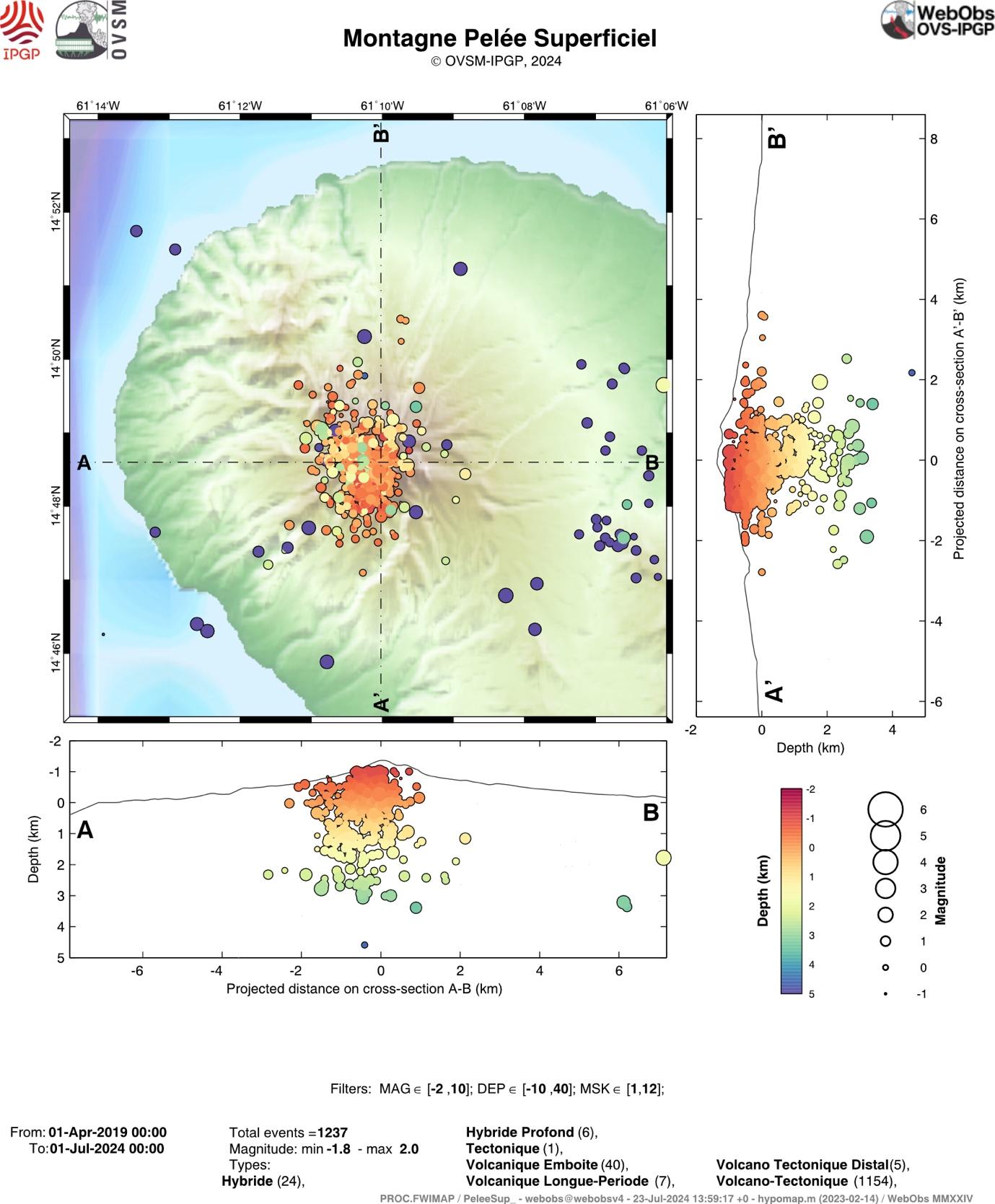
*

***Figure S2.*** *Location of the seismicity recorded on Montagne Pelée by the OVSM-IPGP from April 2019 to July 2024. The seismicity is represented using a map and two profiles: a west to east profile: AB and a south to north profile: A’B’. Hypocenters located at depths between 5 and 40 km are represented on the map as purple circles only. The map was generated using WebObs 2.6.4 operational system*^3^*.*

*
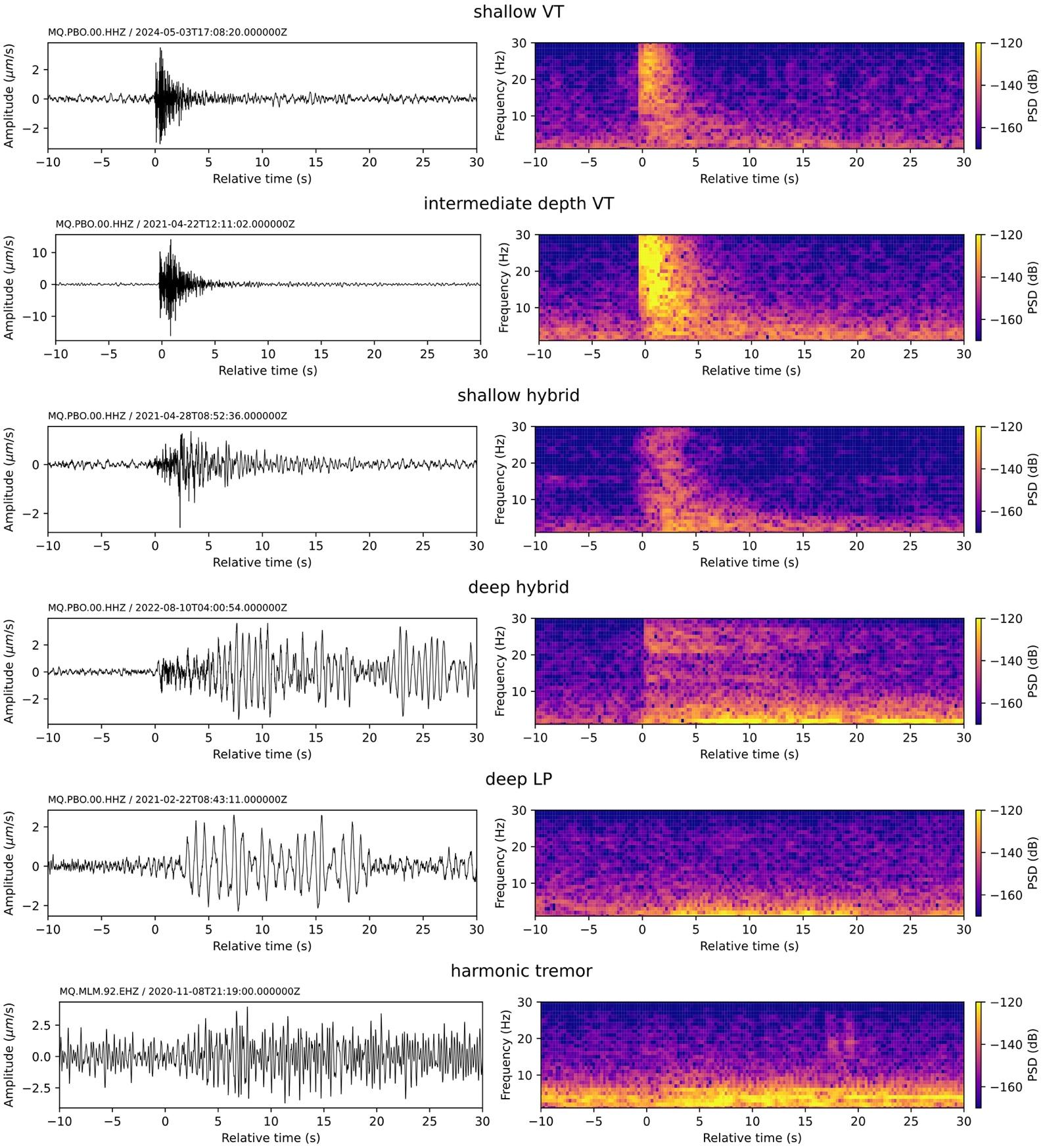
*

***Figure S3.*** *Example of the various seismic event types (shallow and intermediate depth VT, shallow and deep hybrid, deep LP, tremor). We show an example of seismic waveform and spectrogram for each event. Each seismic waveform is obtained after: (i) removing the mean of the seismic record, (ii) applying a 5% cosine taper, (iii) bandpass filtering in the frequency domain the data before deconvolution with four corner frequencies [0.05, 0.1, 45, 50] of the frequency taper, (iv) removing the instrumental response, (v) removing the mean, (vi) applying a 5% Hanning taper, and (vii) bandpass filtering between 1 and 30 Hz with a zero-phase 4-pole Butterworth filter. The power spectral density (PSD) in spectrograms is represented in units of dB related to 1 (m/s)^2^ / Hz.*

*
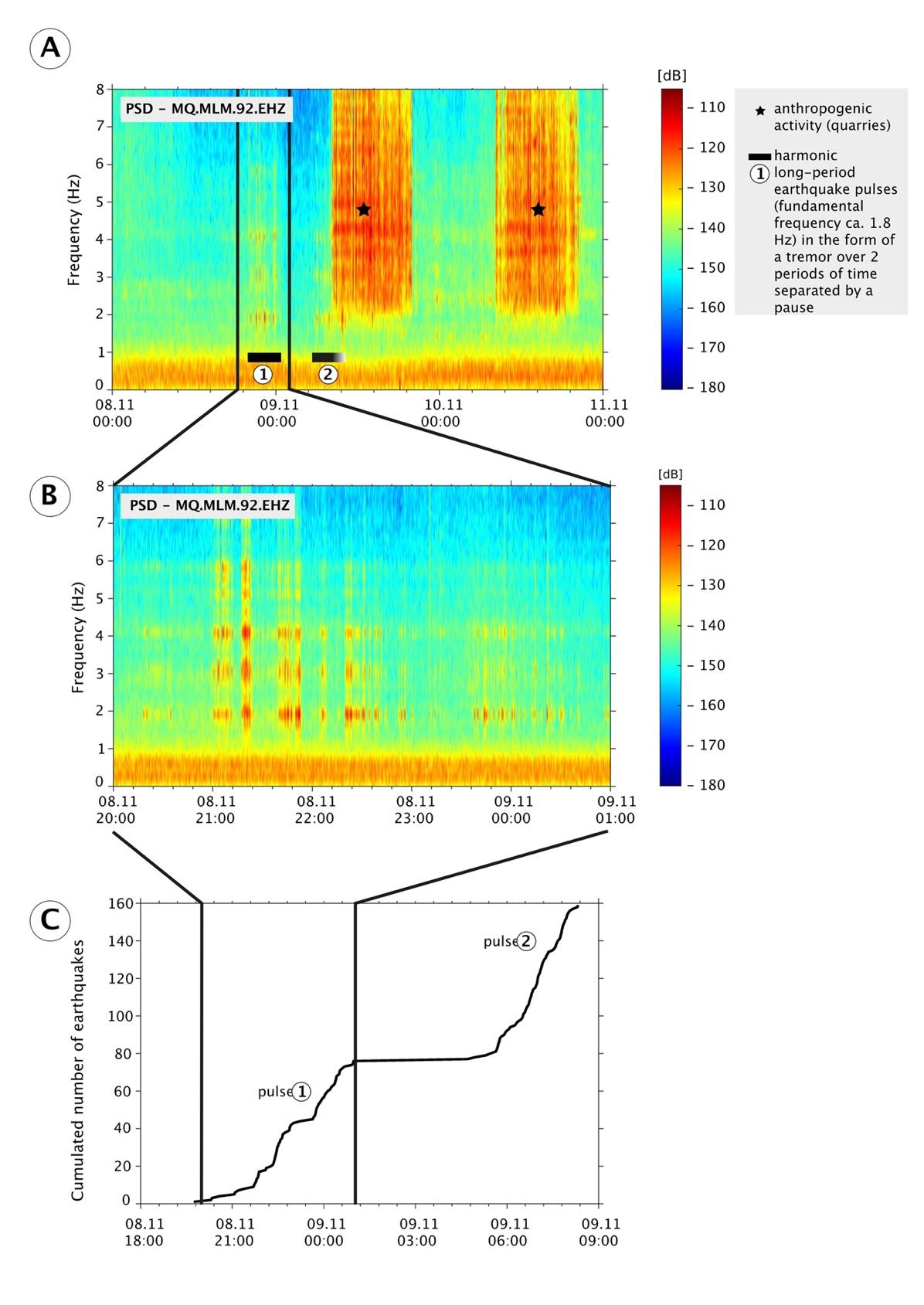
*

***Figure S4.*** *Observations of 159 comparable signals recorded on November 8 and 9, 2020 at the MLM seismic station. (A) Spectrogram from November 8 to 11, 2020, after removing the sensor instrumental response. The PSD is represented in units of dB related to 1 (m/s)^2^ / Hz. (B) Zoom of the spectrogram showing the presence of harmonics at the MLM seismic station. (C) Result of an automatic detection of the same type of seismic signals between November 8 and 9, 2020.*


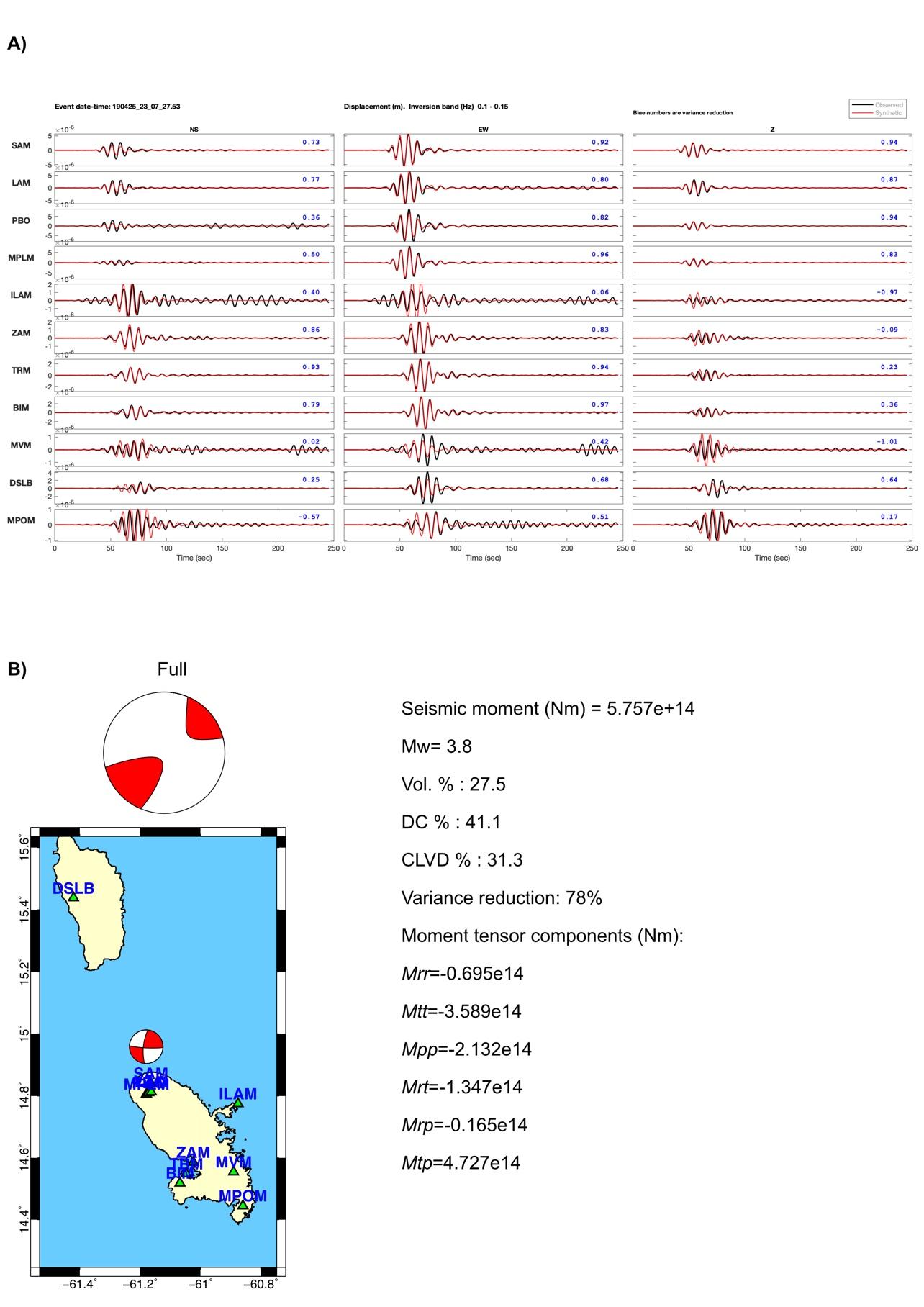


***Figure S5.*** *Results from the full moment tensor (MT) inversion for the April 25, 2019, earthquake at 23:08 UTC. A) comparison between the observed and synthetic waveforms determined from the MT inversion. B) The moment tensor components and the focal mechanism are provided in the standard spherical coordinate system, along with the seismic moment, M_0_ and the moment magnitude, M_W_. The map was produced by the Generic Mapping Tools (GMT)*^4^ *version 4.5.7 and the ISOLated Asperities (ISOLA)*^5^ *graphical user interface (GUI) software package version 5.5.*

*
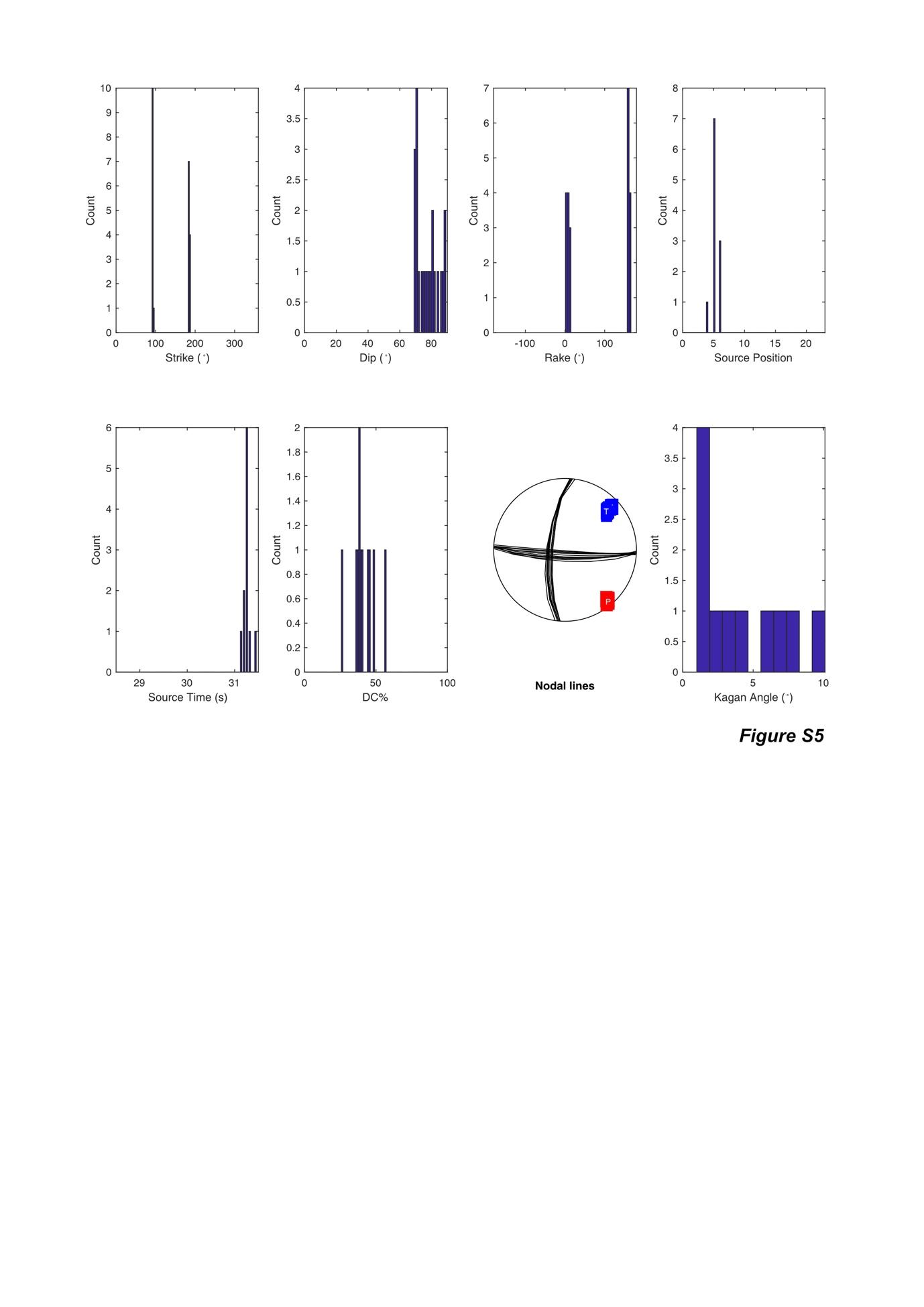
*

***Figure S6.*** *Uncertainties of the results from the full moment tensor inversion were estimated using the jackknife method by repeatedly removing a seismic station. A stable large non-double couple component was obtained. The nodal planes, as well as the pressure (P) and tension (T) axes determined through the jackknife method are similar. The Kagan angles show the double-couple (DC) stability of the acceptable solutions*^5^*.*


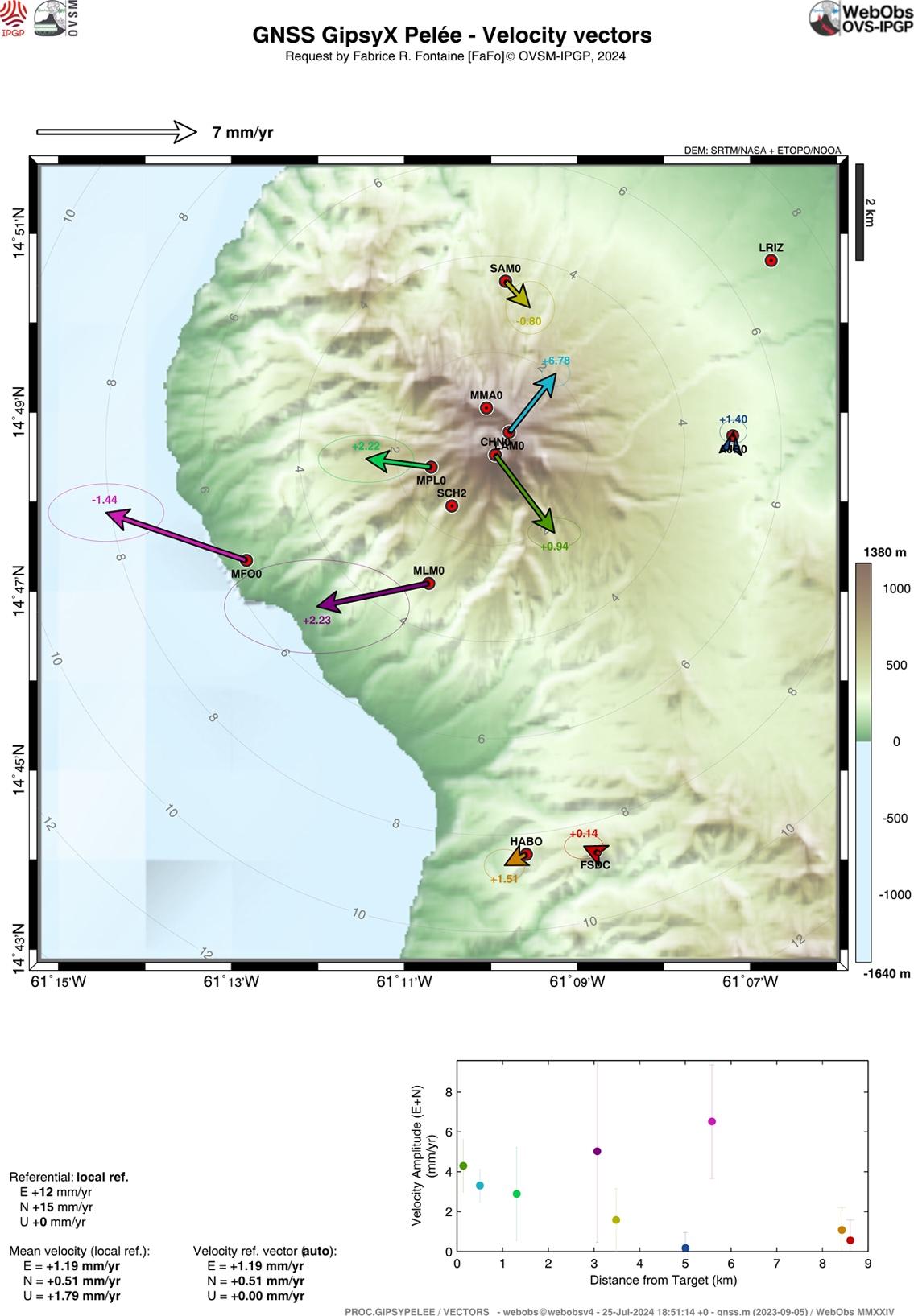



*
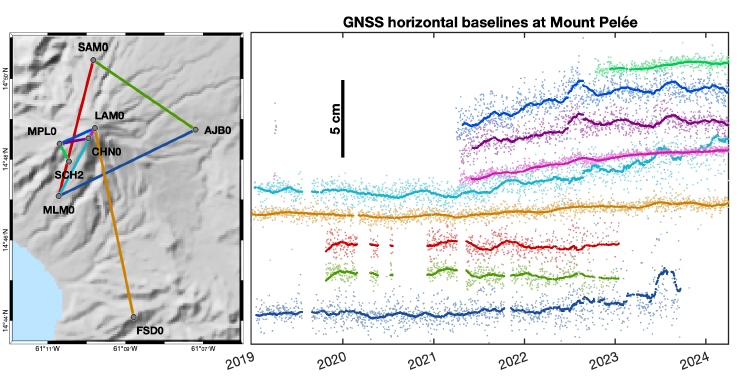
*

***Figure S7.*** *GNSS trend vectors and horizontal baselines at Montagne Pelée. a) GNSS trend vectors from the Montagne Pelée monitoring network from November 1, 2018 to July 1, 2024 from the same period. Data processing and graph produced by WebObs from daily solutions computed by GipsyX/JPL.* *The map was generated using the WebObs 2.6.4 operational system*^3^*. b)* *GNSS variations in horizontal distances (baselines) at Montagne Pelée, calculated from daily data recorded by the permanent GNSS network. (Left) Position of baselines between each pair of stations (FSD0 corresponds to the station located at the former observatory). (Right) Evolution of horizontal distances for each station pair over nearly five years (January 2019 to April 2024): with raw daily data (dots) and values filtered using a 60-day rolling average (solid lines). Periods without data correspond to technical interruptions or stations switched to continuous recording during 2021 (source OVSM-IPGP monthly bulletin April 2024, F. Beauducel /IPGP, https://www.ipgp.fr/wp-content/uploads/2024/05/OVSM_2024_04_fra.pdf).*


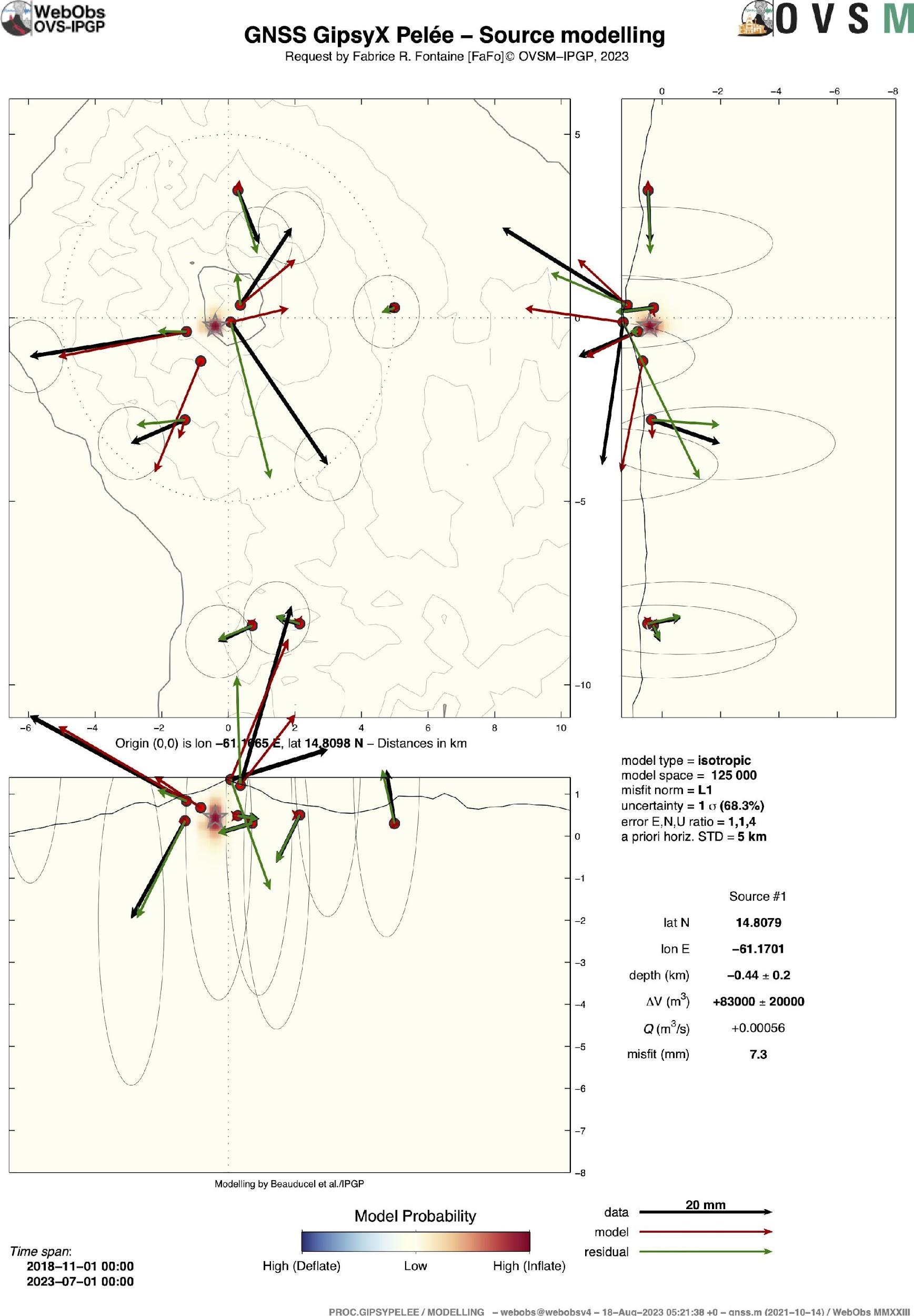

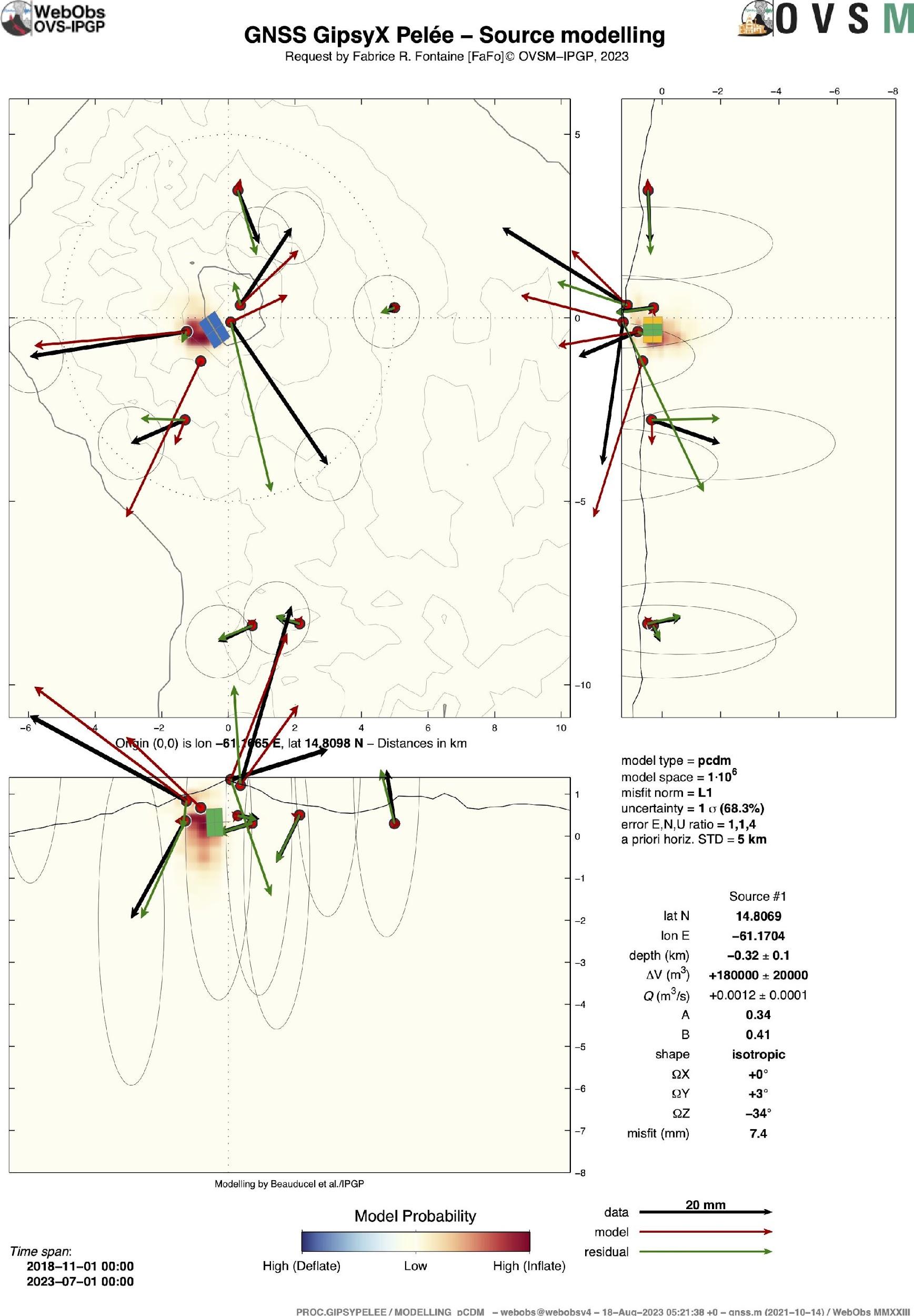


***Figure S8.*** *Results from the modeling of GNSS (Global Navigation Satellite System) displacements from the Montagne Pelée monitoring network from November 2018 to July 2023. a) Position of the source and its volume variation based on Mogi modeling. ΔV is the total volume variation of the source. Q is a flow rate estimation. b) Source location and shape determined using pCDM modeling. ΔV is the total volume variation of the source. A and B are dimensionless shape ratios ranging from 0 to 1 (as defined by equations 2 and 3 in Beauducel et al.*^6^*). Q is a flow rate estimation. ΩX, ΩY and ΩZ are the clockwise angles of rotations around X, Y and Z axes respectively. pCDM dislocation planes are represented in yellow, green and blue. Data processing and graphs were produced by the WebObs operational system.*

*
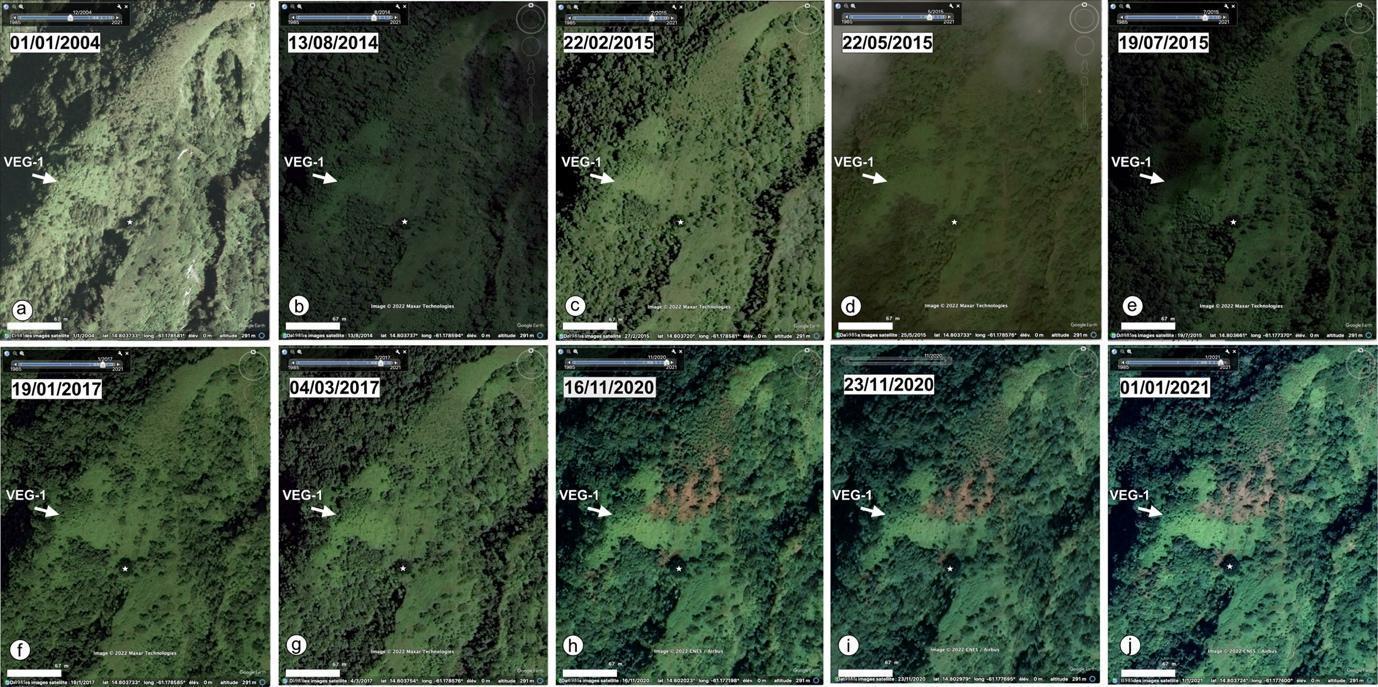
*

***Figure S9.*** *Timeseries of the evolution of the VEG-1 degraded vegetation zone from Google Earth. The authors used Google Earth Pro version 7.3.6.10201 (*[*https://www.google.com/earth/about/versions/#earth-pro*](https://www.google.com/earth/about/versions/#earth-pro)*) to generate the subfigures. Dates: a) January 1, 2004, b) August 13, 2014, c) February 22, 2015, d) May 22, 2015, e) July 19, 2015, f) January 19, 2017, g) March 4, 2017, h) November 16, 2020, i) November 23, 2020, and j) January 1, 2021.*



*
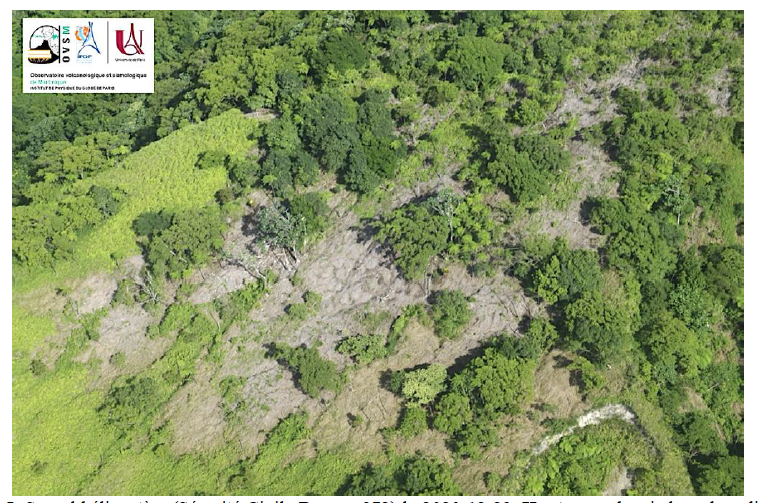
*



*
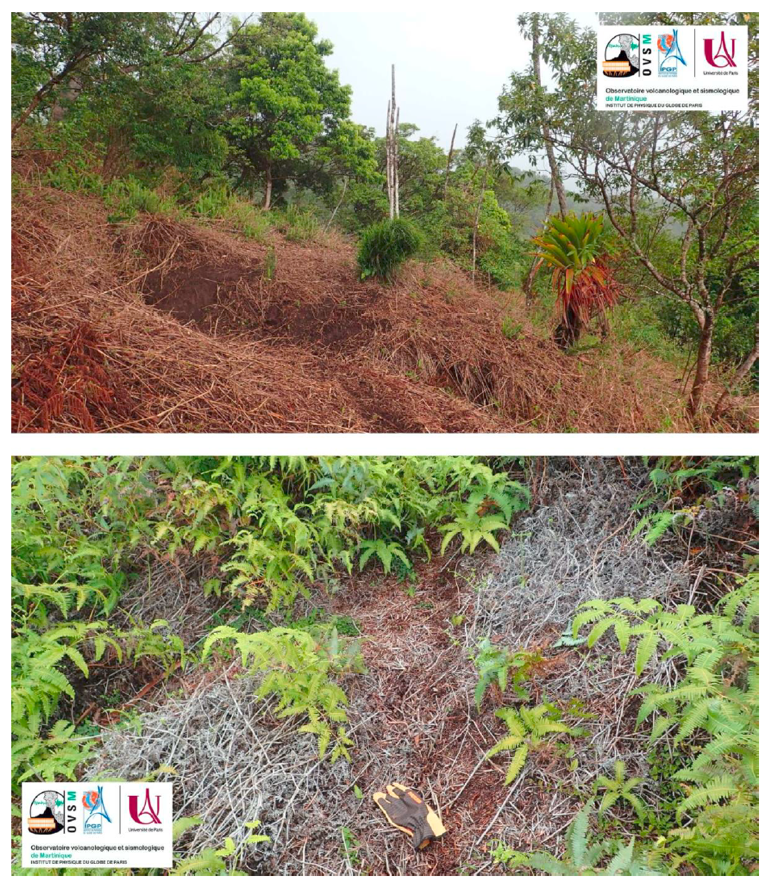
*



***Figure S10.*** *Views of the degraded and vegetation kill zone VEG-1 on the southwest flank of Montagne Pelée (central point of the area approximately 730 m above sea level; latitude 14.80207, longitude -61.17708). See text for details. a) Helicopter survey (Sécurité Civile Dragon 972) on December 29, 2020. Top: view from the southeast towards Morne Plumé, showing in places the total absence of vegetation. (Photo OVSM-IPGP). b)* *View on the ground of the degraded and vegetation kill zone at around 745 m altitude in the central part of* ***Fig. S10a****. Note the presence of totally desiccated vegetation on the ground, the spread of browning upwards, and headless tree ferns. c) View on the ground of the degraded and vegetation kill zone in the upper part of VEG-1 zone, where anomalous CO_2_ concentrations were measured as shown by the purple circle near the top right border of Fig. 4C in the main text). (Photos OVSM-IPGP, taken from OVSM-IPGP Bulletin February 2021).*

*
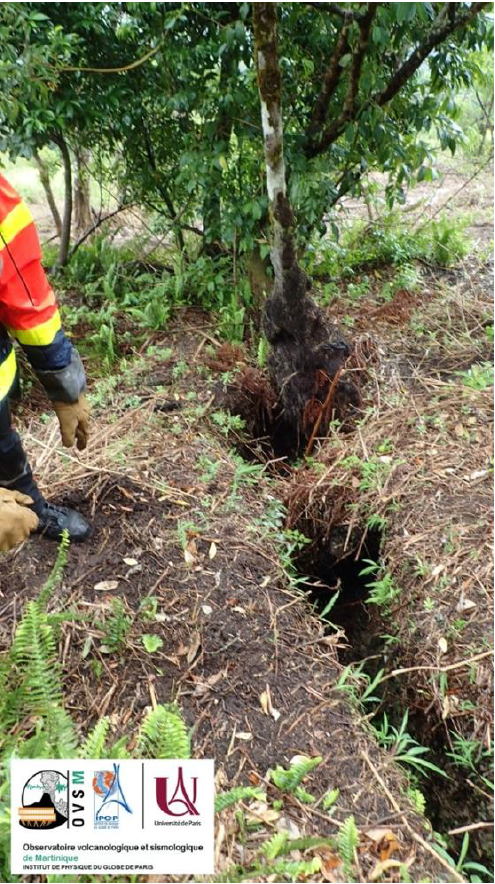
*

***Figure S11.*** *View of an open fracture present at around 740 m in altitude (see* ***Fig. 4c****), situated in the center of an area of browned, degraded, and dead vegetation within the VEG-1 zone. The totally desiccated vegetation has collapsed into the fracture, covering it, showing that the fracture formed after the vegetation had already died. This observation, along with the absence of moss or vegetation on the fracture walls, suggests that it is a recent formation. The fracture width varies between 25 and 60 cm, with a depth of around 2 m. At this site, on February 8, 2021, carbon dioxide (CO_2_) values in the ambient air at 1.5 m above ground level were recorded at up to 0.26% vol. CO_2_, or 2,600 ppm. (Photo OVSM-IPGP; taken from OVSM-IPGP Monthly Bulletin February 2021).*


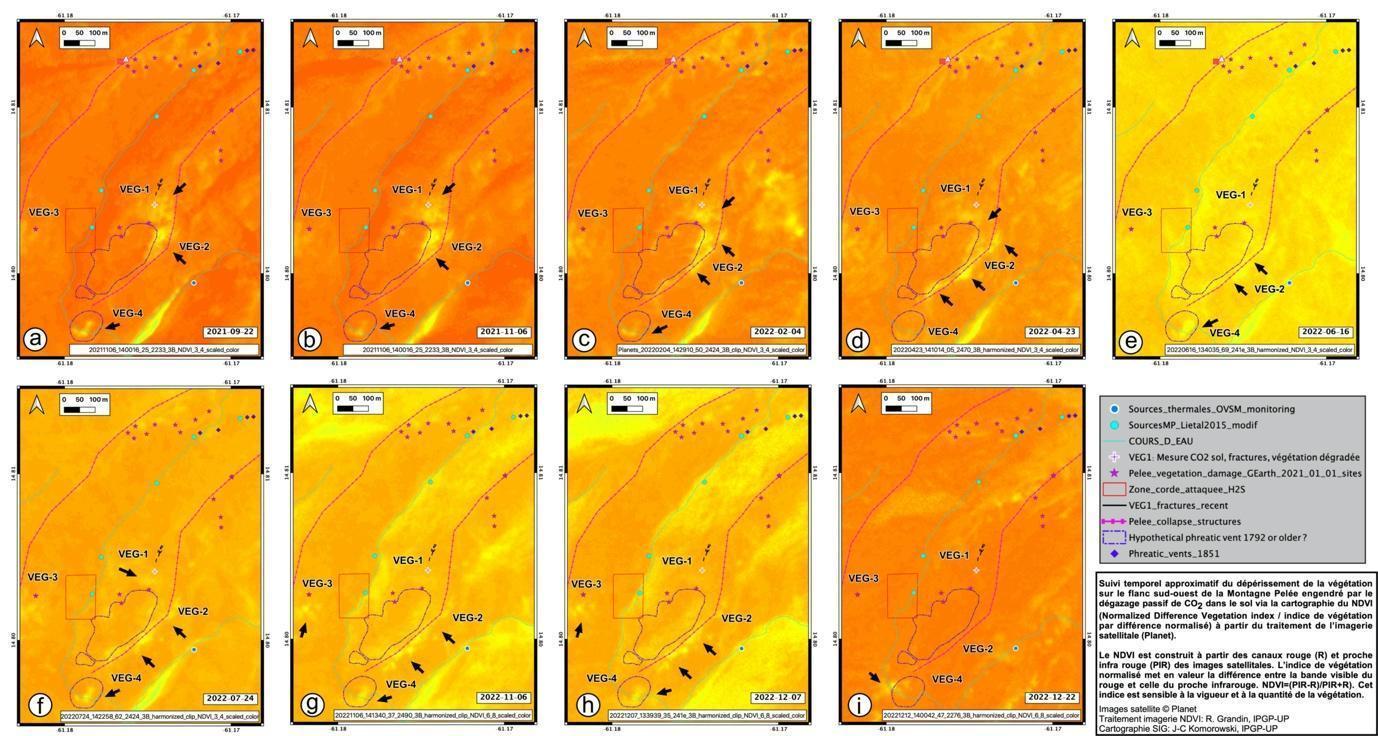


***Figure S12.*** *Timeseries of the evolution of 4 degraded vegetation zones: VEG-1 to VEG-4 from the analysis of the NDVI. Dates: a) September 9, 2021, b) November 6, 2021, c) February 4, 2022, d) April 23, 2022, e) June 16, 2022, f) July 24, 2022, g) November 6, 2022, h) December 7, 2022 and i) December 22, 2022.*

***
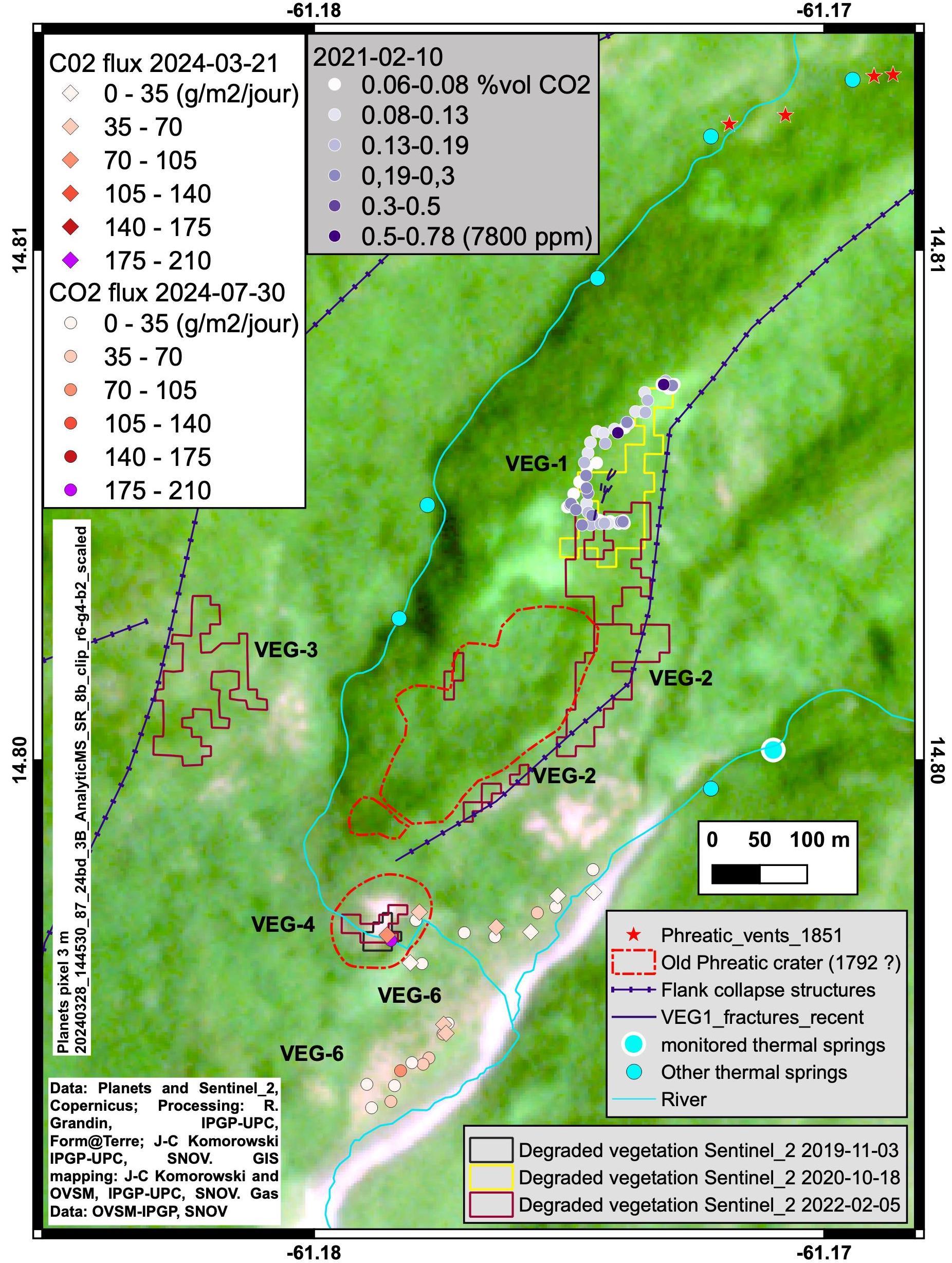
***

***Figure S13.*** *Exploratory and reconnaissance mapping showing the location of flux measurement points for diffuse ground CO_2_ degassing, measured with the WEST Systems accumulation chamber during a heliborne mission with the Martinique Civil Security Dragon 972 on March 21, 2024. The authors created this map using QGIS software version 3.12.0 (https://download.qgis.org/downloads/), incorporating modified Sentinel 2 and Planet images. The maximum flux (at site VEG-6-7) of 84 g/m^2^/day was measured in the crater (zone VEG-4). Areas with a flux >25 g/m^2^/day indicate that the CO_2_ flux necessarily includes a non-biogenic component of volcanic origin, and therefore of magmatic origin*^7^*. These flux measurements confirm that degraded and vegetation kill zones are associated with fluxes of CO_2_ that disrupt the photosynthetic function of vegetation, an effect that can be detected and monitored by satellite images via the NDVI measurement (see above). In the VEG-1 zone, the red and white cross indicates the area where concentrations of up to 5,000 to 7,800 ppm, well above the atmospheric CO_2_ concentration of around 420 ppm, were detected during the first mission to the zone on February 8, 2021 (see OVSM-IPGP Bulletin, February 2021) (Source: OVSM-IPGP, F. Jadelus, D. Melezan, J-G Gabriel, J-C Komorowski and R. Grandin, IPGP) (Taken from OVSM-IPGP Bulletin, March 2024).*

*
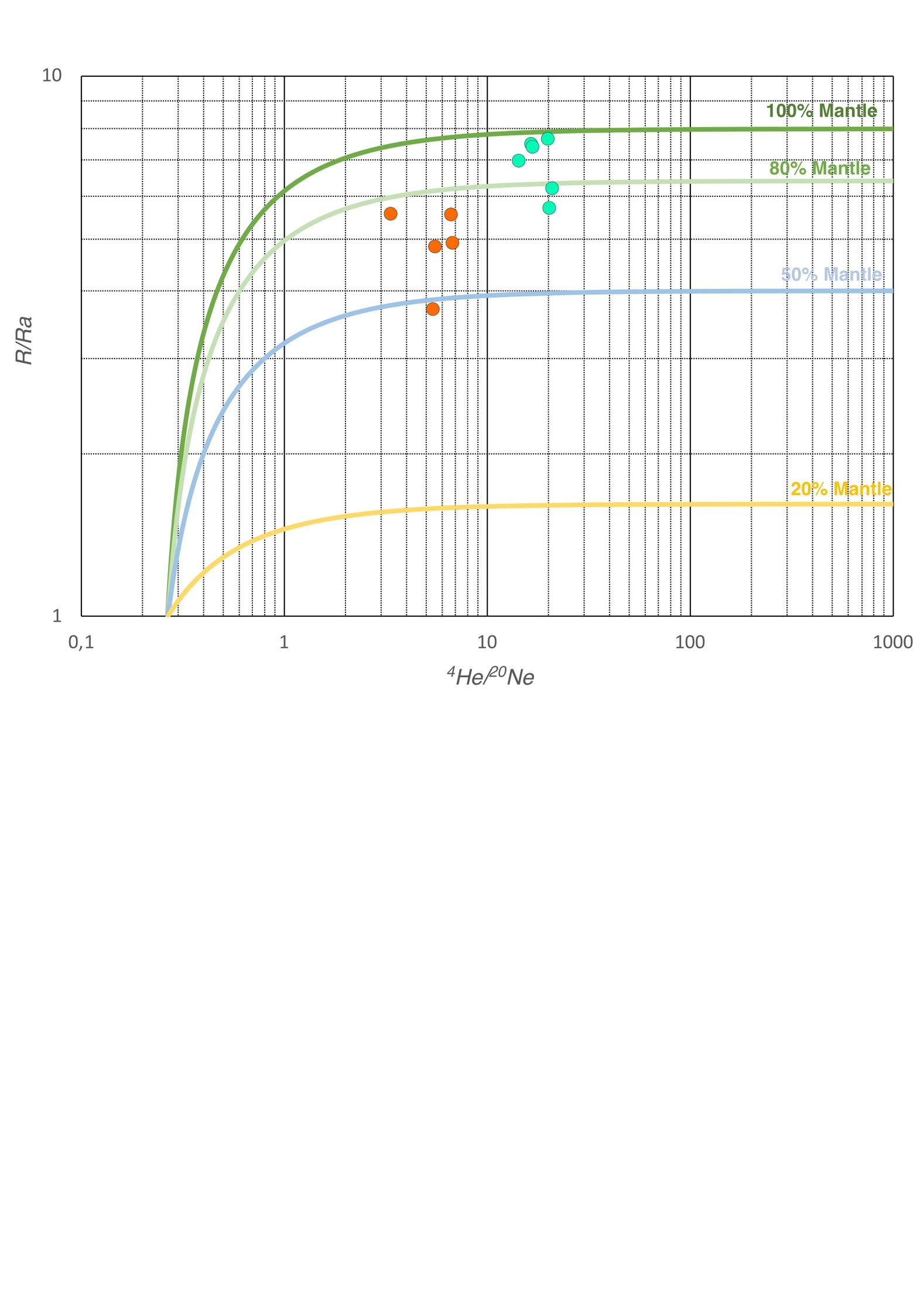
****Figure S14.*** *R/Ra (where R is the ^3^He/^4^He measured in the sample and Ra is the atmospheric ratio) versus ^4^He/^20^Ne for samples of seawater gas from Saint Pierre (Martinique) in red and gas from the thermal spring of the Chaude River on Montagne Pelée in blue. The curves provide results of R/Ra from mixing models between three end-members: the atmosphere, the Mid-Oceanic Ridge Basalt (MORB) and the crust. The proportion of helium coming from the upper mantle (commonly accepted to be represented by the MORB end-member) corresponds to about 70% of the gas withdrawn from seawater in Saint Pierre, and 90% of the gas from the thermal spring of the Chaude River. Crustal helium is more abundant in seawater gas from Saint Pierre (25%) than in gas from the thermal spring of the Chaude River (<10%). 2 to 10% of helium is of atmospheric origin: 2% of gas from the thermal spring of the Chaude River and 4 to 10% for samples of seawater gas from Saint Pierre. Thus, gas samples from seawater are a little more contaminated by the atmosphere than those from thermal spring of the Chaude River.*

*
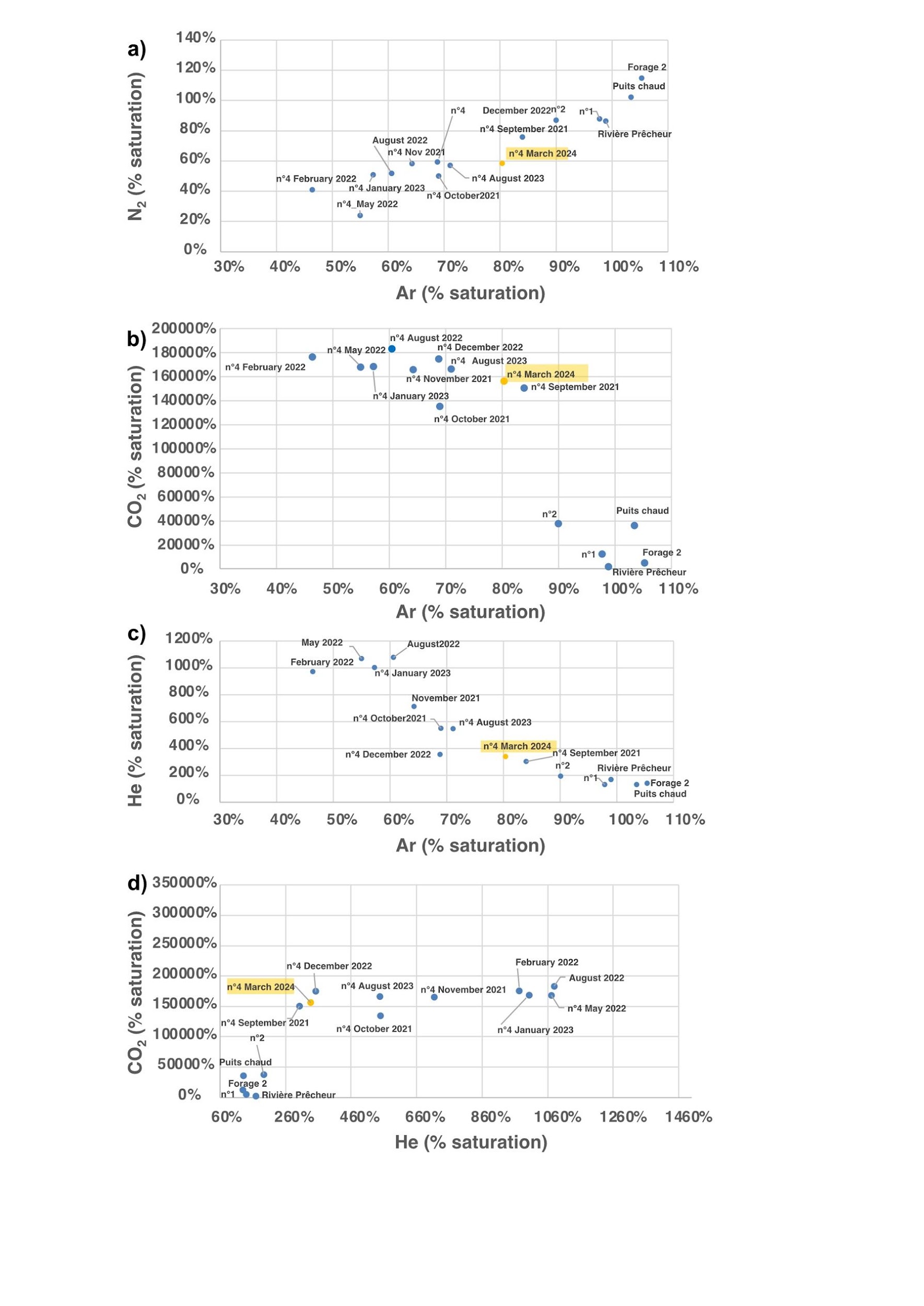
*

***Figure S15.*** *Evolution of dissolved gases in water samples from thermal springs at Montagne Pelée. a) N_2_ versus Ar, b) CO_2_ against Ar, c) He versus Ar and d) CO_2_ against He. The most recent sampling, performed in March 2024, is highlighted in yellow.*

***
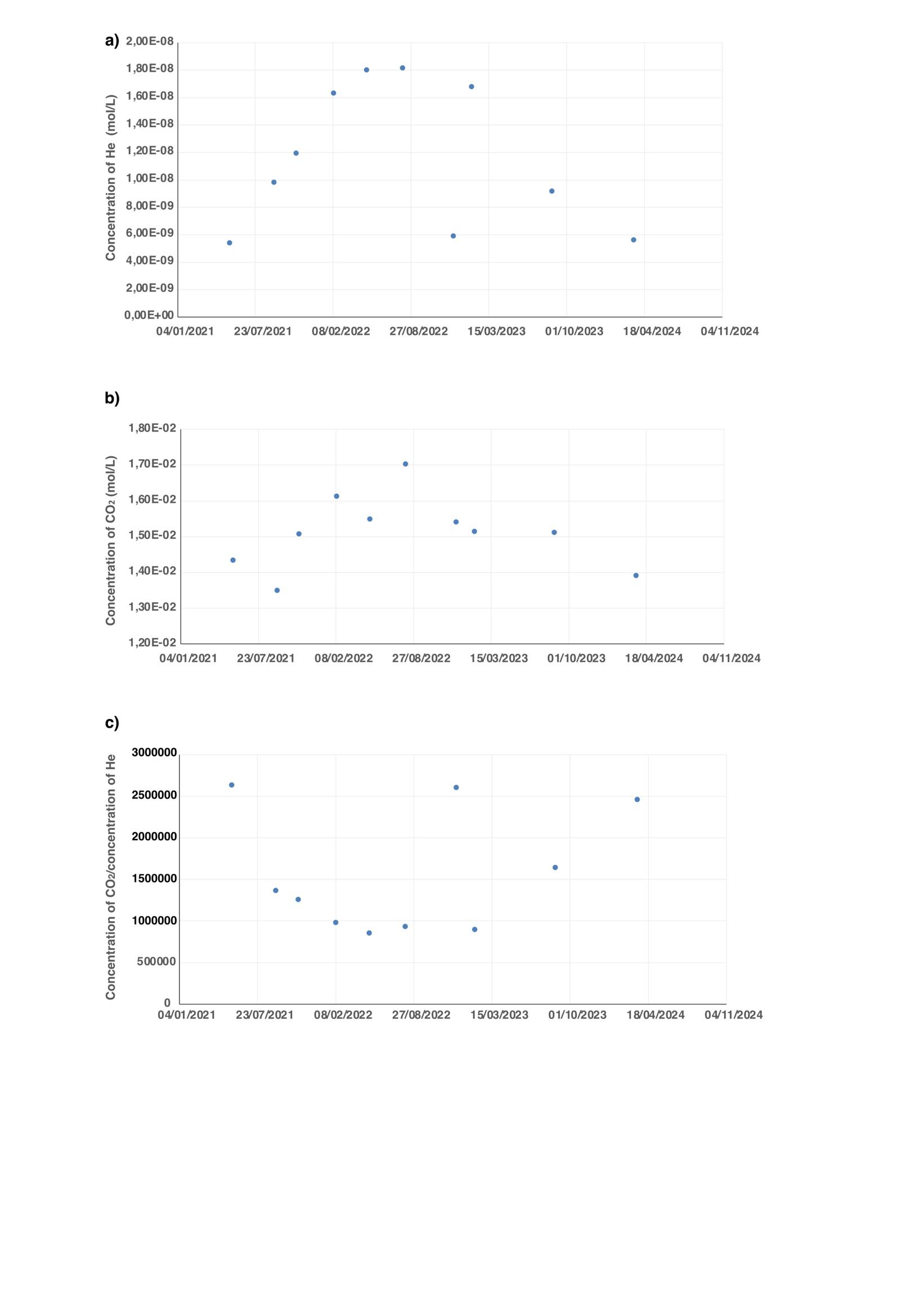
***

***Figure S16.*** *Temporal evolution of He and CO_2_ concentrations at the hottest thermal spring (SC4) of the Chaude River. Temporal variation of He concentration (a) CO_2_ concentration (b) and of the ratio of CO_2_ concentration to the He concentration (c).*

## **Supplementary Tables**

| **Date** | **TECTONIC** (9 < epicentral distance < 21km and depth < 30km) | **TECTONIC**  (9< epicentral distance < 21km and depth < 30km and -90° < back-azimuth < 45°) | **VT** | **TREMOR** | **HYBRID** | **DEEP HYBRID** | **LP** |
| --- | --- | --- | --- | --- | --- | --- | --- |
| **2019-01** | 2 | 2 | 8 | 0 | 0 | 0 | 0 |
| **2019-02** | 7 | 5 | 15 | 0 | 0 | 0 | 0 |
| **2019-03** | 1 | 1 | 13 | 0 | 0 | 0 | 0 |
| **2019-04** | 22 | 21 | 126 | 0 | 0 | 0 | 0 |
| **2019-05** | 8 | 8 | 23 | 0 | 0 | 0 | 0 |
| **2019-06** | 3 | 3 | 7 | 0 | 0 | 0 | 0 |
| **2019-07** | 0 | 0 | 15 | 0 | 0 | 0 | 0 |
| **2019-08** | 5 | 4 | 31 | 0 | 0 | 0 | 0 |
| **2019-09** | 0 | 0 | 64 | 0 | 0 | 0 | 0 |
| **2019-10** | 0 | 0 | 52 | 0 | 0 | 0 | 0 |
| **2019-11** | 9 | 1 | 253 | 0 | 0 | 0 | 1 |
| **2019-12** | 0 | 0 | 95 | 0 | 0 | 0 | 0 |
| **2020-01** | 0 | 0 | 93 | 0 | 0 | 0 | 0 |
| **2020-02** | 0 | 0 | 60 | 0 | 0 | 0 | 0 |
| **2020-03** | 0 | 0 | 59 | 0 | 0 | 0 | 0 |
| **2020-04** | 0 | 0 | 236 | 0 | 0 | 0 | 0 |
| **2020-05** | 1 | 1 | 165 | 0 | 0 | 0 | 0 |
| **2020-06** | 0 | 0 | 63 | 0 | 0 | 0 | 0 |
| **2020-07** | 2 | 0 | 119 | 0 | 0 | 0 | 0 |
| **2020-08** | 0 | 0 | 82 | 0 | 0 | 0 | 0 |
| **2020-09** | 0 | 0 | 212 | 0 | 0 | 0 | 0 |
| **2020-10** | 0 | 0 | 157 | 0 | 0 | 0 | 0 |
| **2020-11** | 0 | 0 | 114 | 1 | 0 | 0 | 0 |
| **2020-12** | 1 | 0 | 454 | 3 | 4 | 0 | 0 |
| **2021-01** | 0 | 0 | 229 | 3 | 2 | 0 | 0 |
| **2021-02** | 3 | 3 | 130 | 2 | 1 | 0 | 1 |
| **2021-03** | 0 | 0 | 147 | 1 | 0 | 0 | 0 |
| **2021-04** | 0 | 0 | 595 | 0 | 10 | 0 | 0 |
| **2021-05** | 0 | 0 | 208 | 0 | 0 | 0 | 0 |
| **2021-06** | 0 | 0 | 128 | 0 | 1 | 0 | 0 |
| **2021-07** | 1 | 0 | 273 | 0 | 1 | 0 | 0 |
| **2021-08** | 0 | 0 | 403 | 0 | 1 | 0 | 0 |
| **2021-09** | 2 | 0 | 421 | 0 | 9 | 0 | 0 |
| **2021-10** | 1 | 0 | 120 | 0 | 0 | 0 | 0 |
| **2021-11** | 0 | 0 | 177 | 1 | 0 | 0 | 0 |
| **2021-12** | 1 | 0 | 97 | 0 | 0 | 0 | 0 |
| **2022-01** | 0 | 0 | 84 | 0 | 0 | 1 | 0 |
| **2022-02** | 0 | 0 | 110 | 0 | 2 | 0 | 1 |
| **2022-03** | 0 | 0 | 35 | 0 | 0 | 0 | 0 |
| **2022-04** | 0 | 0 | 99 | 0 | 2 | 0 | 1 |
| **2022-05** | 5 | 4 | 84 | 0 | 2 | 1 | 1 |
| **2022-06** | 4 | 2 | 12 | 0 | 0 | 0 | 0 |
| **2022-07** | 0 | 0 | 3 | 0 | 0 | 0 | 0 |
| **2022-08** | 0 | 0 | 20 | 0 | 0 | 1 | 0 |
| **2022-09** | 1 | 1 | 43 | 0 | 3 | 1 | 0 |
| **2022-10** | 0 | 0 | 98 | 0 | 2 | 1 | 1 |
| **2022-11** | 0 | 0 | 19 | 0 | 0 | 0 | 0 |
| **2022-12** | 0 | 0 | 43 | 0 | 0 | 2 | 1 |
| **2023-01** | 0 | 0 | 9 | 0 | 0 | 0 | 1 |
| **2023-02** | 0 | 0 | 5 | 1 | 0 | 0 | 0 |
| **2023-03** | 1 | 0 | 25 | 0 | 0 | 1 | 1 |
| **2023-04** | 1 | 1 | 56 | 0 | 1 | 1 | 1 |
| **2023-05** | 0 | 0 | 25 | 0 | 0 | 0 | 0 |
| **2023-06** | 3 | 0 | 8 | 0 | 0 | 0 | 2 |
| **2023-07** | 0 | 0 | 16 | 0 | 0 | 1 | 4 |
| **2023-08** | 0 | 0 | 31 | 0 | 0 | 0 | 0 |
| **2023-09** | 0 | 0 | 25 | 0 | 1 | 0 | 0 |
| **2023-10** | 1 | 0 | 7 | 0 | 0 | 0 | 0 |
| **2023-11** | 0 | 0 | 12 | 0 | 0 | 0 | 0 |
| **2023-12** | 0 | 0 | 14 | 0 | 0 | 0 | 0 |
| **2024-01** | 0 | 0 | 16 | 0 | 0 | 1 | 0 |
| **2024-02** | 1 | 0 | 109 | 0 | 0 | 0 | 0 |
| **2024-03** | 1 | 0 | 71 | 0 | 1 | 0 | 1 |
| **2024-04** | 0 | 0 | 36 | 0 | 0 | 0 | 0 |
| **2024-05** | 1 | 1 | 157 | 0 | 0 | 0 | 0 |
| **2024-06** | 0 | 0 | 9 | 0 | 0 | 0 | 0 |

## ***Table S1.*** *Monthly number of detected VT, tremor, LP, hybrid, deep hybrid, LP and two selections of tectonic earthquakes during the unrest at the Montagne Pelée volcano: 2019 to 2024. We selected tectonic earthquakes occurring in the distance range 9*−*21 km, and at depths shallower than 30 km, as recorded by the OVSM-IPGP from January 1, 2019, to July 1, 2024. The monthly number of tectonic events from this selection, characterized by a back-azimuth in the range -90° to 45°, is also indicated in gray in the third column for comparison.*

| **Name** | **Date of first observations** | **Depth from surface (km)** | **Localisation** |
| --- | --- | --- | --- |
| VT-1 | Recurrent earthquakes since at least the 70s | 0.7 ± 1 | hydrothermal system area, upstream of the Chaude River |
| VT-2 | April 19, 2021 | 2.3-4 | near the Samperre cliff |
| VT-3 | April, 25 2021 | ≃ 2 ± 1 | near the Samperre cliff |
| VT-4 | April 26, 2021 | ≃ 1-2 | near the Samperre cliff |
| VT-5 | June 24, 2021 (01:54:06 UTC) and one earthquake on March 13, 2014 | ≃ 0.5-1 | hydrothermal system area |
| VT-6 | July 1, 2021 and one earthquake on March 11, 2014 | ≃ 1 | hydrothermal system area |
| VT-7 | November 26, 2021 | ≃ 0.7-1.1 | hydrothermal system area |
| VT-8 | November 27 and 28, 2021 | ≃ 6.9-9.5 | near Ajoupa-Bouillon |
| VT-9 | December 5, 2021 | ≃ 7.2-9.4 | near Grand-Rivière |

***Table S2.*** *Different families of VT earthquakes recorded at Montagne Pelée volcano (Martinique) from 2019 to 2024.* *Comparison of the depths of different VT earthquake families recorded at Montagne Pelée and the chronology of these VT earthquakes.*

**Supplementary Note**

**Earthquake swarms during volcanic unrest**

Several types of signals were observed during recent volcanic unrests worldwide. Earthquake swarms occurred in 1992-1995 before the start of the eruption in July 1995 of Soufrière Hills on Montserrat^8^. Earthquake swarms were also described before the explosive activity of Soufrière of St Vincent in 2020−2021^9^. The 2018 unrest phase of La Soufrière of Guadeloupe located at around 132 km northwest of Martinique^10^ was characterized by earthquake swarms, four felt earthquakes (with a maximum felt earthquake of magnitude *M_W_*=3.7), and an increase in fumarolic activity. A swarm of shallow volcano-tectonic (VT) earthquakes and intense ground deformation were also observed three weeks before four explosions during the 2004-2008 eruption of Mount St Helens^11,12^.

**Tectonic activity contemporaneous with volcanic unrest**

Due to its tectonic context, the overriding Caribbean plate in the northern part of the Lesser Antilles regularly experiences tectonic events, mostly occurring in the 8−30 km depth range along arc parallel, en echelon fault systems with mostly normal to strike-slip mechanisms^13^. We selected crustal tectonic events occurring during the last decade at a distance of between 9 and 20 km from the summit of Montagne Pelée, where the 20 km upper limit corresponds to the typical range of distal volcano-tectonic swarms as defined by White and McCausland^12^. We observe a swarm of tectonic events between November 2018 and August 2019, located in the Dominica Channel. These events are situated at a horizontal distance of 16 ± 2 km north-northwest of Montagne Pelée and at depths of 12.5 ± 2.8 km. The largest event (*M_Lv_*=4.1) of this seismic swarm occurred on April 25, 2019. The time at which this swarm began, a few months before the VT earthquakes appeared, the depth of the earthquakes, and their distance from Montagne Pelée are typical of distal earthquakes as defined by White and McCausland^14^. Indeed, just a few months prior and after the onset of the volcanic unrest at Montagne Pelée, the observatory recorded three felt earthquakes during this seismic swarm, which was located in the Dominica Channel (**Fig. 3**), on a fault in transtension with left lateral motion: an *M_Lv_*=3.3 event on December 14, 2018, at 00:55, an *M_Lv_*=4.1 event on April 25, 2019, at 23:08, and an *M_Lv_*=3.5 event on August 22, 2019, at 14:57. These three earthquakes occurred 12−14 km below sea level.

The focal mechanism of the April 25, 2019 felt tectonic earthquake was determined using full moment tensor inversion (see Methods). The moment magnitude calculated for this earthquake is *M_W_* = 3.8 (**Fig. S5**). The source is located between a double couple, a closing crack, and an implosion source type in the *k*-*ε* space proposed by Shuler et al.^15^. Such a mechanism may be related to both a tectonic mechanism and a magmatic process involving closing crack or cavity. Such a large non-double-couple component is a common feature of distal VT seismicity^14^.

These earthquakes occurred on a known left-lateral tectonic fault that has produced earthquakes in the past as recorded by the OVSM-IPGP. However, the increase in activity on this fault just prior to and contemporaneous with the onset of deep seismicity below and near Montagne Pelée and of the shallow VT seismicity is compatible with the model of White and McCausland^16^, who introduced the definition of distal VT earthquakes that are commonly recorded in numerous volcanic unrest and eruptive sequences (cf. for example Montserrat in Figure 7 of White and McCausland^16^). For these authors, distal VT are VT earthquakes occurring on faults 2 to 30 km away from the axis of a magmatic plumbing system below an active volcano that is in a state of unrest often precursory to eruptive activity. The injection of magma from crustal depths into the base of the magmatic plumbing system can exert perturbation of the stress field that can propagate distally and exacerbate the stress cycle on nearby tectonic structures already near rupture conditions that can be released through high frequency seismic activity. Between December 2018 and April 2019, the tectonic earthquakes from the Dominica Channel released 81,450 MJ of seismic energy, around 100 times the energy released by shallow VT earthquakes during all the unrest period. An unknown part of that energy might thus be attributed to the deep-sourced volcanic unrest of Montagne Pelée, given the large non-double-couple component of the main shock of that earthquake swarm (see above).

### Actions accomplished in the context of the yellow alert level

Several actions were undertaken to reinforce and expand the volcano monitoring network. The OVSM-IPGP proceeded with the installation of several seismic and GNSS stations, expanded the monitoring of fluid and gas geochemistry, including submarine fumarole sampling, and implemented a method to track the impact of diffuse CO_2_ soil degassing on the vigor of the vegetation using remote sensing and satellite images. New methods were also elaborated and implemented, such as template detection and machine-learning-based real-time seismicity monitoring^17^. The OVSM-IPGP initiated on December 2020 the publication of weekly report in French and English^18^ that is still ongoing and which is complementary to the monthly bulletin^19^. The OVSM-IPGP expanded its nominal communication and community awareness strategy to develop numerous activities^20–22^ such as: holding public meetings in eight villages located on or near the volcano between December 2020 and April 2021; contributing to a special frequently asked questions webpage by CESECEM^23^, holding several scientific conferences including one held in conjunction with the Mayor of Saint-Pierre on June 30, 2023. The OVSM-IPGP also actively participated in discussions and working sessions with civil protection authorities in Martinique and mainland France and made significant contributions (hazard evaluation) to the new March 2022 revision of the ORSEC volcano crisis response plan of the Préfecture of Martinique^24^. This plan was tested in the first volcano crisis exercise on December 7, 2022, organized by the Préfecture of Martinique, for which the OVSM-IPGP produced a detailed scenario and tested crisis response protocols. OVSM-IPGP staff also actively participate in the Expert committee on volcanology that was newly constituted in 2021 for internal discussions and advice to the Director's office of the Institut de physique du globe de Paris (IPGP), regarding the four active French volcanoes monitored by IPGP.

### Open questions which could be resolved with future data

Can we expect a phreatic phase or magmatic eruption or a cessation of this unrest phase in the next months or years?

Several scenarios are possible as already proposed by previous studies^24–27^ as for instance:

- the end of volcanic unrest,
- a phreatic eruption and the onset of a fumarolic activity and then the end of the volcanic unrest,
- a fumarolic activity, then a phreatic eruption phase, then pyroclastic flows and then the end of the eruption,
- a fumarolic activity then a phreatic eruption phase then a lateral blast followed by a Plinian explosion phase,
- a fumarolic activity then phreatic eruption phase then a lateral blast and a landsliding debris avalanche,
- a fumarolic activity then phreatic eruption phase and then a lateral blast and dome forming eruption.

A quantitative assessment of the likelihood of any of these scenarios is fraught with significant epistemic and aleatory uncertainty. What changes might a major tectonic earthquake (magnitude ≥7) occurring in the vicinity of Montagne Pelée produce on the volcanic system, which is currently in a state of unrest and disequilibrium? Feuillet et al.^13^ showed that a mechanical coupling exists between volcanic activity and moderate to large historical earthquakes in this region. Although the monitoring network was considerably upgraded in 2012 and in 2021, an uncertainty still remains, albeit reduced, on the ability to detect eruptive precursors, particularly for phreatic eruptions or the onset of fumarolic activity. Do deep signals (seismicity, low-frequency events, and CO_2_ degassing) constitute the telltale yet subtle signs of the onset of gas-rich fluid injection into the deep magma storage zones of the Montagne Pelée transcrustal magmatic system, albeit of limited volume or at a limited flux rate currently not sufficient to trigger the destabilization of the deep magma mush?

The apparent correlation between the Dominica Channel seismic events and the volcanic unrest is perhaps the most important observational point. One might say, for example, that magma migration and tectonic earthquakes within the Caribbean plate both fundamentally are related to the stress field within the plate, which fluctuates as subduction proceeds. Therefore, it is sensible to look at both volcanic unrest and tectonic events as potentially two aspects of a single process. The two "end-member" possibilities are: a) that a tectonic event has perturbed the volcano and particularly its hydrothermal system, but not with enough energy to trigger magma ascent or eruptive phenomena; and b) that some magma migration has indeed occurred due to the induced stress changes, increasing the likelihood of some kind of eruptive event. Further work on the fundamental phenomena is required to progress on this question.

Research in the past decades has shown that active volcanic systems behave according to three embedded timescales: 1) magma genesis over timescales on the order of hundreds of thousands to millions of years; 2) the destabilization of eruptible magma present in zones of the mush magma lenses of transcrustal magmatic systems on the order of less than a month to several months or a few years^25^ ; and 3) the final ascent of magma that can take place on timescales on the order of minutes to a few days^28–31^. At Montagne Pelée, Boudon et al.^32^ showed that the magma of the 1902 eruption took a minimum of 15 days for its final ascent from its shallow storage depth of 6-9 km to the surface. Magma ascent during Plinian eruptions is even faster. Given that the transit time of magma that could be involved in a future eruption at Mount Pelée is unknown, these rapid timescales must be taken into consideration in the tracking and analysis of ongoing unrest by civil protection authorities.

**References**

1. Beauducel, F. *et al.* Rapport d’activité 2023 de l’Observatoire volcanologique et sismologique de Martinique, Institut de physique du globe de Paris, 1-83, (2024).

2. Hirn, A., Girardin, N., Viodé, J.-P. & Eschenbrenner, S. Shallow seismicity at Montagne Pelée volcano, Martinique, Lesser Antilles. *Bull. Volcanol.* **49**, 723–728 (1987).

3. Beauducel, F. *et al.* WebObs: The Volcano Observatories Missing Link Between Research and Real-Time Monitoring. *Front. Earth Sci.* **8**, 1–22 (2020).

4. Wessel, P. & Smith, W. H. F. Free software helps map and display data. *Eos Trans. Am. Geophys. Union* **72**, 441–446 (1991).

5. Sokos, E. & Zahradník, J. Evaluating centroid-moment-tensor uncertainty in the new version of ISOLA software. *Seismol. Res. Lett.* **84**, 656-665, (2013).

6. Beauducel, F., Peltier, A., Villié, A. & Suryanto, W. Mechanical Imaging of a Volcano Plumbing System From GNSS Unsupervised Modeling. *Geophys. Res. Lett.* **1–9**, (2020).

7. Viveiros, F. *et al.* Soil CO2 emissions at Furnas volcano, São Miguel Island, Azores archipelago: Volcano monitoring perspectives, geomorphologic studies, and land use planning application. *J. Geophys. Res.* **115, B12208**, (2010).

8. Aspinall, W. P. *et al.* Soufrière Hills Eruption, Montserrat, 1995–1997: Volcanic earthquake locations and fault plane solutions. *Geophys. Res. Lett.* **25**, 3397–3400 (1998).

9. Joseph, E. P. *et al.* Responding to eruptive transitions during the 2020–2021 eruption of La Soufrière volcano, St. Vincent. *Nat. Commun.* **13**, (2022).

10. Moretti, R. *et al.* The 2018 unrest phase at La Soufrière of Guadeloupe (French West Indies) andesitic volcano: Scrutiny of a failed but prodromal phreatic eruption. *J. Volcanol. Geotherm. Res.* **393**, (2020).

11. Scott, W. E., Sherrod, D. R. & Gardner, C. A. Overview of the 2004 to 2006, and Continuing, Eruption of Mount St. Helens, Washington, Chapter 1. in *A Volcano Rekindled: The Renewed Eruption of Mount St. Helens, 2004-2006* (eds. Sherrod, D. R., Scott, W. E. & Stauffer, P. H.) (U.S. Geological Survey Professional Paper 1750, 2008).

12. Bergfeld, D., Evans, W. C., Spicer, K. R., Hunt, A. G. & Kelly, P. J. Evidence for degassing of fresh magma during the 2004–2008 eruption of Mount St. Helens: Subtle signals from the hydrothermal system. *J. Volcanol. Geotherm. Res.* **343**, 109–121 (2017).

13. Feuillet, N., Beauducel, F. & Tapponnier, P. Tectonic context of moderate to large historical earthquakes in the Lesser Antilles and mechanical coupling with volcanoes. *J. Geophys. Res.* **116**, (2011).

14. White, R. & McCausland, W. Volcano-tectonic earthquakes: A new tool for estimating intrusive volumes and forecasting eruptions. *J. Volcanol. Geotherm. Res.* **309**, 139–155 (2016).

15. Shuler, A., Ekström, G. & Nettles, M. Physical mechanisms for vertical-CLVD earthquakes at active volcanoes. *J. Geophys. Res.* **118**, 1569–1586 (2013).

16. White, R. A. & McCausland, W. A. A process-based model of pre-eruption seismicity patterns and its use for eruption forecasting at dormant stratovolcanoes. *J. Volcanol. Geotherm. Res.* **382**, 267–297 (2019).

17. Retailleau, L. *et al.* A Wrapper to Use a Machine‐Learning‐Based Algorithm for Earthquake Monitoring. *Seismol. Res. Lett.* (2022) doi:10.1785/0220210279.

18. Observatoire volcanologique et sismologique de Martinique, Institut de physique du globe de Paris. Bilans hebdomadaires de la Montagne Pelée. (2024).

19. Observatoire volcanologique et sismologique de Martinique, Institut de physique du globe de Paris. Bulletins mensuels. (2024).

20. Fontaine, F. R. *et al.* Global Volcanism Program, Report on Pelee (France). *Bull. Glob. Volcanism Netw.* **46**, (2021).

21. Fontaine, F. R. *et al.* Rapport d'activité 2021 de l'Observatoire volcanologique et sismologique de Martinique, Institut de physique du globe de Paris, 1-81, (2022).

22. Komorowski, J.-C. *et al.* The ongoing multiparameter volcanic unrest at Mount Pelée (Martinique, France): challenges for scientific, monitoring and civil protection response, Cities on volcanoes COV12 international conference, IAVCEI, Antigua, Guatemala, 11-16 February 2024, abstract. (2024).

23. Conseil Economique, Social, Environnemental de la Culture et de l’Education de Martinique (Césécem). 10 réponses sur la situation actuelle de la Montagne Pelée. (2021).

24. Préfecture de Martinique. Dispositif départemental ORSEC, Phénomènes volcaniques Montagne Pelée, Service interministériel de Défense et de Protection Civiles (SIDPC). 1-110 , (2022).

25. Boudon, G. & Balcone-Boissard, H. Volcanological evolution of Montagne Pelée (Martinique): A textbook case of alternating Plinian and dome-forming eruptions. *Earth-Sci. Rev.* **221, 103754**, (2021).

26. Cheminée, J. L., Boudon, G., Dagain, J., Rançon, J. P. & Traineau, H. Volcanic hazard in the French Antilles. in *Natural Risk and civil protection* (eds. Horlick-Jones, E., Amendola & Casale), 96-115 (Published by E & FN Spon., 1995).

27. Michaud-Dubuy, A., Carazzo, G. & Kaminski, E. Volcanic hazard assessment for tephra fallout in Martinique. *J. Appl. Volcanol.* **10**, 8, (2021).

28. Costa, F., Shea, T. & Ubide, T. Diffusion chronometry and the timescales of magmatic processes. *Nat. Rev. Earth Environ.* **1**, 201–214 (2020).

29. Metcalfe, A. *et al.* Magmatic Processes at La Soufrière de Guadeloupe: Insights from Crystal Studies and Diffusion Timescales for eruption onset. *Front. Earth Sci.* **9**, (2021).

30. Metcalfe, A. *et al.* Corrigendum: Magmatic Processes at La Soufrière de Guadeloupe: Insights from Crystal Studies and Diffusion Timescales for eruption onset. *Front. Earth Sci.* **9**, (2021).

31. Berthod, C. *et al.* The 2018-ongoing Mayotte submarine eruption: magma migration imaged by petrological monitoring. *Earth Planet. Sci. Lett.* **571**, (2021).

32. Boudon, G., Balcone-Boissard, H., Villemant, B. & Morgan, D. J. What factors control superficial lava dome explosivity? *Sci. Rep.* **5**, 1–14 (2015).
